# Creating healthy habits for Maryland preschoolers (CHAMP): a cluster-randomized controlled trial among childcare centers

**DOI:** 10.1186/s12966-025-01824-6

**Published:** 2025-12-10

**Authors:** Alysse J. Kowalski, Bridget Armstrong, Angela C.B. Trude, Raquel Arbaiza, Amber Czinn, Laura L. Bellows, Susan L. Johnson, Yan Wang, Erin R. Hager, Maureen M. Black

**Affiliations:** 1 Department of Pediatrics, University of Maryland School of Medicine, Baltimore, MD USA; 2 Department of Population, Family and Reproductive Health, Johns Hopkins Bloomberg School of Public Health, Baltimore, MD USA; 3 Department of Exercise Science, Arnold School of Public Health, University of South Carolina, Columbia, South Carolina, USA; 4 Department of Nutrition and Food Studies, School of Culture, Education, and Human Development, New York University Steinhardt, New York, NY USA; 5 School of Medicine, Faculty of Medical & Health Sciences, Tel Aviv University, Tel Aviv, Israel; 6 Division of Nutritional Sciences, Cornell University, Ithaca, NY USA; 7 Department of Pediatrics, Section of Nutrition, University of Colorado Anschutz Medical Campus, Aurora, CO USA; 8 Department of Prevention and Community Health, District of Columbia, Washington, USA; 9 RTI International, Research Triangle Park, Research Triangle Park, NC USA

**Keywords:** Dietary, Fruit and vegetable consumption; motor skills, Obesity prevention, Physical activity, Willingness-to-try-new-foods

## Abstract

**Background:**

Risks for chronic health problems are embedded in preschoolers’ dietary and physical activity habits. Childcare centers are a potential venue to establish healthy habits. We hypothesized that health-promoting center plus parent interventions would improve preschoolers’ dietary and physical activity outcomes, including body weight, over control.

**Methods:**

We made local modifications to the 30-week Food Friends^®^ curriculum to develop a center intervention, Creating Healthy Habits Among Maryland Preschoolers (CHAMP), and a parent website (CHAMP+), aligned with the CHAMP intervention. The CHAMP intervention included a manual, web-based lessons plans, handouts, resources, and program materials implemented by CHAMP-trained staff. We evaluated effectiveness in a 3-arm cluster randomized controlled trial. Childcare centers serving low-income communities were recruited (2017–2020) from 10 counties and randomized to center (CHAMP), center plus parent (CHAMP+), or Control arms. Willingness-to-try-new-food, fruit and vegetable (FV) preference, motor competence (Test of Gross Motor Development-2), moderate-vigorous-physical-activity (MVPA, 7-day accelerometry), and anthropometry (BMI z-scores) were measured at baseline/endline (6 months post-baseline) by assessors masked to intervention status. Linear mixed models examined differences in changes among arms. Center baseline nutrition/physical activity environmental quality (Environment and Policy Assessment and Observation) was examined as moderating intervention effects.

**Results:**

Fifty-six centers were randomized (CHAMP = 21, CHAMP+ = 20, Control = 20); 855 children. Centers were diverse by location, race, and income; children were mean age 48.44 (SD 7.50) months, 54% male; 26% experienced overweight/obesity. Analyses adjusted for baseline differences in child age, race, and ethnicity. The intervention improved motor competence (gross motor quotient: pooled CHAMP/CHAMP+ vs. Control 5.67 [95% CI: 0.60, 10.75]; locomotor score: pooled CHAMP/CHAMP+ vs. Control 1.74 [95% CI: 0.43, 3.05]) and reduced BMIz (pooled CHAMP/CHAMP+ vs. Control (-0.08 [95% CI: -0.15, 0.00]); with no intervention effects on willingness-to-try-new-foods, FV preference, or MVPA and no impact enhancement by the parent intervention (CHAMP+). Moderation analyses showed stronger increases in willingness-to-try-new-foods and MVPA in centers with higher quality nutrition/physical activity environments.

**Conclusions:**

Childcare center interventions can improve motor competence and reduce BMIz among preschoolers. Higher quality nutrition/physical activity environment can increase the impact of interventions on children’s dietary behaviors and physical activity, contributing to healthy habits.

**Trial registration:**

NCT03111264.

**Supplementary Information:**

The online version contains supplementary material available at 10.1186/s12966-025-01824-6.

## Background

The preschool years are a critical period as children’s dietary and physical activity behaviors become habits that can influence dietary and physical activity behaviors throughout life [1, 2]. In the United States (U.S.), many preschoolers are not meeting recommendations for fruit and vegetable intake [3, 4] or for 60 min of daily moderate-vigorous physical activity (MVPA) [5]. These patterns are consistent with patterns reported among preschoolers globally with low fruit and vegetable consumption [6] and low adherence to 24-hour movement guidelines [7]. Taken together, if poor dietary and physical activity behaviors become habits, they may impact not only preschoolers’ current health, but the risk of excess weight gain and lifelong chronic diseases [8, 9].

The increasing prevalence of obesity among U.S. preschoolers (5–12.7% from 1970 to 2020) [3, 10] emphasizes that effective multi-component (dietary and physical activity) interventions are needed to reverse the trend and to promote healthy dietary and physical activity habits among preschoolers. In addition to multi-component interventions, interventions implemented at multiple levels of the bio-ecological model [11] are needed to build preschoolers’ dietary and physical activity habits. Individual factors, such as pickiness and neophobia, hesitancy to try novel foods [12] and gross motor competence, (e.g., ball handling and running) [13, 14] are associated with dietary and physical activity behaviors, respectively. Environmental factors also contribute to preschoolers’ habits, influenced by parents’ consumption of fruits and vegetables and engagement in physical activities [15]. Although studies have begun to examine the context where interventions occur (e.g., home, childcare, and healthcare) [16], the ecological quality of environments and the mechanisms whereby policies, systems, and the environment, including staff engagement influence the formation of preschoolers’ dietary and physical activity habits have been understudied [17, 18].

In the U.S., over 6 million preschoolers attend weekly out-of-home care, with 83% in center-based care. Globally, childcare centers are expanding rapidly, as women are increasingly in the work force [19]. Thus, childcare settings are a potential venue for promoting healthy dietary and physical activity habits [20]. However, intervention-driven changes in fruit and vegetable consumption and physical activity have been inconsistent across childcare-based studies, with reliance frequently on changes in body mass index (BMIz), few direct measures of children’s dietary or physical activity practices, and limited attention to the ecological quality of childcare centers [21].

To assess the feasibility of implementing nutrition promotion programs in childcare centers, we conducted a pilot randomized trial in 22 childcare centers [22]. The nutrition-based curriculum delivered to childcare center directors and weekly nutrition-based activities delivered to children by center staff (including teachers, aides, and directors) were based on Social Learning Theory [23]. The children learned by observing others and being surrounded by healthy options. The intervention was effective in improving the quality of the nutrition environment. However, children’s fruit and vegetable consumption was not altered, suggesting the need for a more engaging, multi-component and multi-level intervention [22].

To address this, we implemented the CHAMP intervention (Creating Healthy Habits Among Maryland Preschoolers) by making local modifications to The Food Friends^®^ curriculum [24]. We selected The Food Friends^®^ curriculum, developed at Colorado State University, because it has been shown to successfully increase preschoolers’ willingness-to-try-new foods and improve gross motor performance [24–28]. The Food Friends^®^ curriculum actively engages children through activities involving “super heroes”, such as Bella Bean (Supplemental Material 1–2). Thus, children may be more likely to develop motivation and self-efficacy, consistent with the tenets of Social Cognitive Theory [23]. To extend the intervention, we added a parent website with wellness-promoting information, aligned with the CHAMP intervention: the CHAMP+ intervention (Supplemental Material 3). The parent website, built on the principles of Social Cognitive Theory, engaged parents in the same topics and activities that the children were experiencing in childcare [29].

The aims of this study were to evaluate the impact of a multi-component (diet and physical activity), multi-level (center and parent) trial among childcare centers on preschoolers’ dietary and physical activity behaviors and anthropometrics. The center-based intervention, referred to as CHAMP, was integrated into daily interactive lessons delivered by childcare staff. The parent intervention, CHAMP+ extended the center-based intervention to parents through web-based diet and physical activity activities that aligned with the center-based intervention. Centers that received the parent intervention (CHAMP+) also received the center intervention (CHAMP). We tested one a priori hypothesis and an exploratory hypothesis that examined the moderating effects of the quality of the nutrition/physical activity environment [24]:

1. Children in CHAMP+ centers (center and parent interventions) would demonstrate more improvement on primary outcomes (willingness-to-try-new-food, gross motor competency, and physical activity) and on secondary outcomes (fruit and vegetable preference and less weight gain) compared to children in Control centers. Children in centers that received the center-based intervention, but not the parent intervention (CHAMP centers) would be in an intermediate position (CHAMP+ > CHAMP > Control).
2. Exploratory. The impact of the center intervention (CHAMP and CHAMP+ pooled) compared to Control on willingness-to-try-new-foods and physical activity would vary by the nutrition/physical activity environmental quality of the childcare centers, with stronger effects in higher quality environments.

## Methods

### Study design

#### Evaluation

We enrolled childcare centers to participate in a 3-arm cluster randomized controlled trial to evaluate the CHAMP, CHAMP+, or waitlist Control intervention. The protocol [24] was registered at clinicaltrials.gov (NCT03111264) and approved by the University of Maryland School of Medicine Institutional Review Board (HP-00065933) prior to the recruitment of the centers or children. Parents (or legal caregivers) provided written consent for themselves and their child prior to randomization; children provided verbal assent. A Data and Safety Monitoring Board met annually to review study progress and monitor for adverse events. Results are reported in accordance with the CONSORT extension for cluster randomized controlled trials [30].

#### Eligibility, recruitment, randomization, and evaluation design

Centers were eligible if they were: licensed, enrolled children ages 3–5 years, served low-income communities, and were < 50 miles from the university. Serving low-income communities was defined as participating in the Child and Adult Care Food Program, honoring childcare vouchers, or charging less than $300/week. Head Start centers were excluded due to existing wellness policy infrastructure [31]. Using a list of licensed centers from the state Office of Childcare, centers were recruited over three years (2017–2018, 2018–2019, 2019–2020) with each center participating for one academic year (September through May).

Treatment was assigned using a stratified cluster randomization procedure. Using center-level data, centers were organized into strata matched on location (urban, suburban, rural), size (< 50 vs. *≥* 50 children, race (proportion Black, White, Other) and ethnicity (proportion Hispanic, non-Hispanic, Other). Within each stratum, centers were randomly allocated to CHAMP (center), CHAMP+ (center plus parent) or Control, using a computer-generated random number and conducted by a researcher unaware of intervention assignment. All centers had an equal non-zero probability of being assigned to any arm. Randomization was conducted in the Fall of each study year after center recruitment and baseline.

The evaluation was designed to follow the academic year, with a Fall baseline evaluation implemented shortly after recruitment, a midline in mid-Winter, and a Spring endline evaluation. The midline included a subset of the measures. Due to timing and logistical demands, we were unable to collect the midline evaluation for all centers. Thus, the endline evaluation of all interventions occurred in the Spring, introducing a 12-week delay in the evaluation of the Fall intervention. The COVID-19 pandemic disrupted intervention delivery and evaluation for the 2019–2020 cohort. This manuscript reports on the findings of objectively measured child outcomes at baseline and endline. Measures administered to parents and staff related to feeding practices have been reported previously [32–35], and will be the subject of subsequent manuscripts, along with additional child measures.

#### Child/parent eligibility

The CHAMP intervention was delivered to all children in participating centers. Each center identified a liaison (i.e., teacher or staff member) who informed families about the study, collected consent forms, and facilitated communication with the research team. Eligibility for the evaluation cohort included: age 3–5 years, English speaking, attended childcare ≥ 3 days/week, and intended to attend through the spring. Exclusion criteria included developmental delays, significant food allergies, or behavior problems that prevented children from participating in the evaluation tasks. Children with physical disabilities interfering with motor activities, and otherwise eligible, were included, but not assessed for motor competence or physical activity.

#### Sample size estimation

We assumed an average intraclass correlation of 0.03 across child outcomes (willingness-to-try-new-foods [36], fruit and vegetable preference [37], moderate-vigorous physical activity [MVPA] [38], gross motor competence, and body mass index z-score [BMIz] [39]); correlation between repeated measures of 0.50; 80% power; and 20% loss-to-follow-up. Assuming enrollment of 864 children across 54 centers (16 children/center), we had power to detect a small effect (Cohen’s *d* = 0.27) for each outcome [24]. The detected difference between the groups would be equal to 0.27 standard deviations for each outcome, yielding an estimated 7% greater probability of trying a novel food [22], 0.68 and 0.86 point increase in the number of fruits and vegetables rated as “yummy” respectively, 10-minute difference in MVPA, 6-point increase in gross motor percentile score [40], and 0.30 difference in BMIz [41].

#### CHAMP intervention

To develop the CHAMP intervention, we adopted two modules from The Food Friends^®^ curricula (referred to as Mighty Moves and New Foods) to the Mid-Atlantic context by incorporating local food and terminology. We retained the Food Friends^®^ materials [22], with the exception of substituting local food to be tasted. Mighty Moves was an 18-week program (4 activities/week) implemented in the fall/winter that focused on building gross motor skills. New Foods was a 12-week program (2 activities/week) implemented in the winter/spring focused on nutrition. Additional information on Mighty Moves and New Foods can be found in Supplemental Materials 1–2. These interactive lessons (15–20 min) were incorporated into the daily activities and delivered by childcare staff.

The research team trained childcare staff before implementing Mighty Moves and before implementing New Foods. Training (2–3 h) included demonstrations and practice opportunities prior to implementation of the intervention and weekly check-ins during intervention delivery. Staff received a manual and web-access to lessons plans, handouts, resources, and program content (i.e., music) (Supplemental Material 1–2). The research team provided additional training when there were staff changes. The research team used Instacart and personal delivery to provide pre-cut, portioned foods for taste-testing.

#### CHAMP+ intervention

CHAMP+ centers received the childcare center-based intervention plus an educational website for parents with infographics, short videos, and weekly content on children’s diet, physical activity, and wellness activities [29]. CHAMP+ centers received a study tablet to enable staff to take photos of children engaging in the activities. The research staff uploaded photos onto the parent website. The CHAMP+ parent activities were aligned with center activities with the intention of extending center activities so parents. We encouraged parents to engage with their children on topics and activities presented in the Center, consistent with Social Cognitive Theory [23]. Parents were directed to the study website with a unique login per childcare center and alerted weekly of new content by text or email, including uploads of photos of their children engaged in intervention activities, along with monthly challenges and quizzes. Supplemental Material 3 includes additional information on the parent educational website for CHAMP+.

Centers randomized to the Control arm received intervention materials following the endline evaluation, to be implemented the following year. They received no intervention during the study year.

### Process evaluation

To monitor the implementation of the intervention, the research team conducted a weekly phone check-in with each CHAMP and CHAMP+ center. The center staff reported the children’s attendance, the number of lessons implemented, and any challenges regarding the implementation. In addition, the research team made at least two in-person visits to each CHAMP and CHAMP+ center to observe at least two lessons. The research team noted the quality of the lessons including adherence to the objectives, use of materials, staff responses to the children, children’s engagement and responses, and challenges. The information was recorded and shared with the center through a feedback and discussion session. Fidelity across the centers was measured by a mean of 78% of the learning objectives being met. At the conclusion of the intervention, the research team conducted qualitative interviews with the staff, asking them to comment on the curriculum, implementation of the lessons, perceived impact on the children, and recommendations for improvement. The teachers’ primary concern was the lack of time and space to implement the lessons. Detailed findings from the process evaluations are the focus of a separate paper.

### Objective child measures

Fall baseline and Spring endline evaluations were conducted in childcare centers by trained data collectors who were unaware of the center’s randomization arm. Training was conducted for each measure until the data collectors demonstrated competence in administering and scoring each measure. For the Test of Gross Motor Development-2 and the Environmental and Policy Assessment and Observation, data collectors had to meet specific criteria using a gold standard video or senior data collector, as noted below. We used the Qualtrics survey platform to ensure that data collection procedures were systematic and that responses were recorded and range checked to reduce errors. Booster training was conducted prior to each round of data collection.

#### Willingness-to-try-new-foods (primary)

Children received a flight of 6 novel foods and 3 familiar foods (whole wheat cracker, grape, cheese) and were given 10 min to try as many foods as they wished [42, 43]. Novel foods (edamame, papaya, chickpea, water chestnut, olive, and kidney bean) ranged from sweet to savory and were defined from pilot data indicating that the food was unknown to at least 75% of children. A data collector recorded whether each food was tried (touched with the tongue or placed in the mouth) or refused [42, 44]. Willingness-to-try-new-foods was calculated as the proportion of novel foods tried.

#### Fruit and vegetable preference (secondary)

The Fruit and Vegetable Preference assessment was used to assess children’s fruit and vegetable preference [45]. Children were shown individual photographs of 9 fruits and 11 vegetables and asked to rate their preference for each item (yucky, just OK, yummy). Affirmative responses were summed separately for fruits and vegetables.

#### Gross motor skill (primary)

The Test of Gross Motor Development-2 (TGMD-2) includes locomotor and object control subscales [46]. For each subscale, 12 skills (e.g., ball throwing and catching, running, hopping) were rated “1” (accurate completion) or “0” (non-completion). Skill scores were summed and converted to standard scores for locomotor, object control, and gross motor quotient. The TGMD-2, data collectors had to achieve a reliability Kappa > 0.75 against a gold standard video [47].

#### Physical activity (primary)

Actical accelerometers (Philips Respironics, Minimiter, Bend, OR) were secured to the lateral malleolus of the child’s non-dominant ankle and worn for 7 consecutive days [48]. The accelerometers were worn continuously (24 h) and not removed for sleeping or bathing. Movement data were collected in 15-sec intervals (Actical software version 3.11). Partial days and days with implausibly low step counts (e.g., 80 steps/day) were removed from analyses. Age-specific thresholds were applied to calculate daily minutes of MVPA, light, and sedentary activity/sleep [48]. Accelerometers were explained during the consenting/assenting process and caregivers or children could refuse. Data were available on 685 of 855 children (80%).

#### Anthropometry (secondary)

Height was measured to the nearest 0.10 cm with a portable stadiometer (Shorr Productions, Olney, Maryland) following a standardized procedure [49] and was repeated until the difference between measures was < 1.00 cm. Weight was measured to the nearest 0.10 kg with TANITA BWB-800 (TANITA, Tokyo, Japan) digital scales and repeated until two measures were identical. Anthropometry values were used to calculate BMIz based on age- and sex-specific 2000 CDC Growth Charts [50].

### Childcare center measures

The childcare center measures were collected at baseline.

#### Demographic measures

Demographic measures were collected from childcare center records and baseline surveys administered to childcare centers and parents. Child age and sex were obtained from center records. Child race (Asian, Asian Vietnamese, Black or African American, Mixed Race, Native American/American Indian/Alaskan Native, Native Hawaiian or Pacific Islander, White or Caucasian) and ethnicity (Is your child’s ethnicity Hispanic) were collapsed into three categories for analysis (non-Hispanic White, non-Hispanic Black, and all other Hispanic and non-Hispanic races). Household income (percentage of the federal poverty level [% FPL]) was calculated from parent-reported household income and family size [51], and was used to calculate mean household income/center. Centers were classified as urban, suburban, or rural [52] and other center characteristics included size, children’s race and ethnicity, family income, and Child and Adult Care Food Program participation.

#### Moderator: Environment and Policy Assessment and Observation (EPAO)

The EPAO is a comprehensive, objective measure of childcare nutrition and physical activity environmental quality that was collected only at baseline [53]. The measure includes a full-day center observation and document review (food menus, guidelines, and policies) and was assessed at baseline. Items were scored 0 to 3 points reflecting “worst” to “ideal” and averaged within each subscale. Subscales were summed, with higher scores reflecting higher quality nutrition (range: 0–21) and physical activity (range: 0–36) environments. Data collectors were trained using a practice video and had to be cleared for in-person data collection after achieving a reliability of Kappa > 0.8 against a gold standard video provided by the EPAO developer [54].

### Statistical analysis

The data were analyzed using intent-to-treat principles. We retained all participants except those centers that dropped out prior to baseline. We conducted initial descriptive analyses to examine balance on center- and child-level characteristics between arms. Where there was imbalance, we conducted bivariate associations and adjusted models for characteristics associated with outcomes.

For each Hypothesis 1 outcome, we specified a linear mixed model with random intercepts for childcare center and child. Time was treated categorically. To evaluate Hypothesis 1, we specified an interaction between intervention and time to assess differences in the rate of change among arms. We estimated the change in outcome from baseline within each arm and compared the change among arms using pairwise comparisons. Where there was no difference in the change in outcome between the two intervention arms, we repeated this analysis pooling CHAMP and CHAMP+.

For Hypothesis 2, we conducted an exploratory analysis, specifying a three-way interaction between intervention, time, and EPAO score to assess if effectiveness on nutrition-sensitive outcomes (willingness-to-try-new-foods) and physical activity-sensitive outcomes (MVPA) differed by center nutrition and physical activity environmental quality, respectively. Results were visualized by plotting the estimated treatment effect at three levels of center environmental quality (low = −1 SD, moderate = mean, and high = + 1 SD).

#### Sensitivity analysis

Siblings were eligible to enroll in the study (*n* = 65). To account for the increased similarity between children from the same household, we conducted a sensitivity analysis, selecting one sibling from each household at random.

Alpha was set at *p* < 0.05 for Hypothesis 1 and *p* < 0.10 for Hypothesis 2, given that 3-way interactions have lower power in analyses [55]. Analyses were conducted in R v4.3.1 (Vienna, Austria) [56].

## Results

**Fig. 1:**
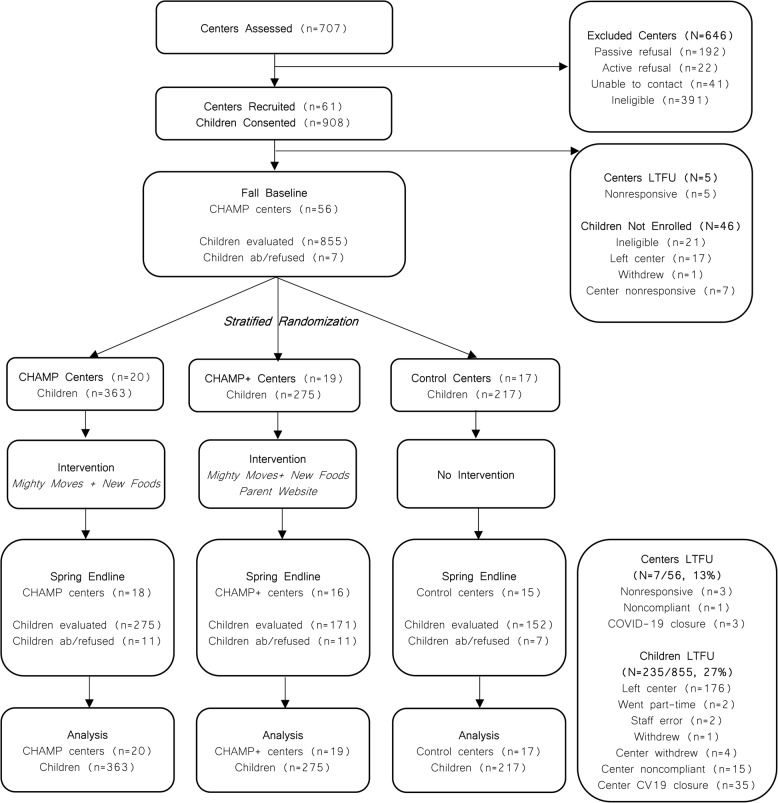
CHAMP cluster-randomized controlled trial CONSORT diagram Abbreviations: ab = absent; CV19 = COVID-19; LTFU = lost to follow-up

We recruited 61 childcare centers across 10 counties. Five centers withdrew before baseline. Fifty-six centers were randomized; four centers withdrew mid-study and three were disrupted by the COVID-19 pandemic. We retained their baseline data, but endline data were not collected. Retention from Fall baseline through Spring endline was 87.5% (49/56) for centers and 72.7% (627/862) for children; 56 centers and 855 children were included in the analysis (Fig. 1).

Centers that withdrew tended to be smaller, have lower incomes, and include predominantly Black children (Supplemental Table 1). Children lost to follow-up were more likely to be enrolled in the CHAMP+ arm and centers with predominantly Black children, lower incomes, and lower quality nutrition and physical activity environments (Supplemental Table 2).

### Baseline childcare center characteristics

Centers were diverse with respect to location, race, and income (Table 1). The majority (71%) were small (≤50 children) and most were suburban (61%), with 29% urban, and 11% rural. We defined racial and ethnic predominance as >70% of the children. Forty-nine percent of centers were predominantly Black, 33% predominantly White, and 18% no predominant race. Mean income varied; 54% of centers had mean family incomes >300% of the FPL, 27% had incomes 185-300% FPL, and 20% had incomes <185% FPL with similar center nutrition and physical activity environments across arms.

**Table 1:** Baseline childcare center and preschooler characteristics in the CHAMP cluster-randomized controlled trial^1^

	CHAMP	CHAMP+	Control
**Center Characteristics**	**n = 20**	**n = 19**	**n = 17**
Mean child participants/center	19.10 (11.05)	15.47 (8.82)	13.24 (7.95)
Center size, ≤50 children	15 (75%)	12 (67%)	12 (71%)
CACFP participation	10 (50%)	7 (41%)	7 (41%)
Children’s race and ethnicity			
>70% White	8 (40%)	5 (28%)	5 (29%)
>70% Black	9 (45%)	11 (61%)	7 (41%)
No group >70%	3 (15%)	2 (11%)	5 (29%)
Mean center income, % federal poverty line	270.6 (97.26)	257.58 (138.1)	314.86 (82.19)
Locale			
Rural	1 (5%)	3 (16%)	2 (12%)
Suburban	16 (80%)	7 (37%)	11 (65%)
Urban	3 (15%)	9 (47%)	4 (24%)
Observed nutrition environment score (EPAO range: 0-21)	8.18 (1.65)	7.87 (1.3)	7.51 (1.25)
Observed physical activity environment score (EPAO range: 0-36)	12.05 (2.94)	11.36 (2.8)	10.97 (1.86)
**Preschooler Characteristics**	**n = 363**	**n = 275**	**n = 217**
Age, months	48.18 (7.62)	47.71 (7.33)	49.8 (7.36)
Gender, male	190 (52%)	158 (57%)	116 (53%)
Race and ethnicity			
non-Hispanic White	224 (63%)	109 (41%)	103 (48%)
non-Hispanic Black	93 (26%)	130 (49%)	67 (31%)
all other^2^	38 (11%)	28 (10%)	43 (20%)
Willingness-to-try-new-foods, % novel foods tried	35% (36%)	34% (34%)	29% (34%)
Fruit and Vegetable Preference			
Number of fruits rated yucky	1.98 (1.79)	2.13 (1.99)	2.27 (1.99)
Number of fruits rated ok	1.87 (2.21)	1.48 (2.03)	1.69 (2.17)
Number of fruits rated yummy	4.92 (2.56)	5.09 (2.55)	4.88 (2.53)
Number of vegetables rated yucky	3.77 (2.95)	3.8 (3.22)	3.82 (2.95)
Number of vegetables rated ok	2 (2.6)	1.61 (2.4)	1.97 (2.54)
Number of vegetables rated yummy	4.93 (3.23)	5.19 (3.42)	4.83 (3.09)
Gross Motor Skill			
Gross motor, standard score^3^	123.45 (13.09)	121.69 (15.99)	123.1 (14.74)
Locomotor, standard score^3^	14.05 (3.12)	13.52 (3.98)	14.5 (3.5)
Object control, standard score^3^	13.75 (2.48)	13.63 (2.57)	13.16 (2.37)
Physical Activity			
MVPA, mins/d	76.88 (27.76)	74.55 (29.57)	77.66 (31.2)
LPA, mins/d	367.7 (50.19)	366.32 (51.98)	363.08 (47.29)
Sed/S, mins/d	995.41 (62.13)	999.13 (67.72)	999.26 (62.04)
BMIz	0.42 (1.05)	0.41 (1.15)	0.45 (0.99)
Weight Status, overweight or obese	95 (26%)	77 (28%)	51 (24%)

*Abbreviations:* *SD* standard deviation, *CACFP* Child and Adult Care Food Program, *EPAO* Environment and Policy Assessment and Observation, *MVPA* moderate to vigorous physical activity, *LPA* light physical activity, *Sed/S* sedentary time/sleep, *BMIz* body mass index z-score

^1^ Presented as mean (SD) or n (%), as appropriate

^2^ Child all other race and ethnicity includes individuals from the following groups: non-Hispanic multiracial (n = 55); Hispanic of any race (n = 29); non-Hispanic other race (n=24); unknown race and ethnicity (n=1)

^3^ 2017-2018 cohort only

### Baseline child characteristics

Preschoolers mean age at enrollment was 48.44 (SD 7.50) months, 54% male, 52% non-Hispanic White, 35% non-Hispanic Black, and 13% other race and ethnicity (7% non-Hispanic multiracial, 3% Hispanic of any race, 3% non-Hispanic other race, and 0.1% unknown). CHAMP had a larger proportion of non-Hispanic White preschoolers (63% compared to 41% CHAMP+ and 48% Control) and Control preschoolers were slightly older.

On average, children were willing to try 33% (SD 35%) or 2 of 6 novel foods. Preschoolers rated 23% (mean 2.10 SD 1.91) fruits and 34% (mean 3.79 SD 3.03) vegetables as yucky and 55% (mean 4.96 SD 2.55) fruits and 45% (mean 4.99 SD 3.25) vegetables as yummy. Mean daily minutes of MVPA and sedentary time/sleep were 76.37 (SD 29.21) and 997.54 (SD 63.81), respectively; 74% met physical activity guidelines of ≥60 minutes MVPA/day. Mean gross motor quotient was 122.74 (SD 14.74); 86^th^ percentile. Most preschoolers were healthy weight with 26% overweight or obese.

### Intervention effects

Intervention effects adjusted for baseline differences in child age and race and ethnicity are shown in Table 2 and results of the pooled CHAMP and CHAMP+ analysis are in Supplemental Table 3. Unadjusted results and sibling sensitivity results are in Supplemental Tables 4 and 5, respectively and were consistent with adjusted findings; therefore, only adjusted results are described. Across all outcomes, intraclass correlations ranged from 0 to 0.064 for center and 0.105 to 0.927 for participants (Supplemental Table 6).

**Table 2:** Baseline to endline changes in objective child measures within and between study arms in the CHAMP cluster-randomized controlled trial

	CHAMP	CHAMP+	Control	CHAMP vs. Control	CHAMP+ vs. Control	CHAMP+ vs. CHAMP
	Mean ∆ (95% CI)^1^	Mean ∆ (95% CI)^1^	Mean ∆ (95% CI)^1^	Adjusted Estimate (95% CI)^2^	Adjusted Estimate (95% CI)^2^	Adjusted Estimate (95% CI)^2^
Willingness-to-try-new-foods, *n* = 757	0.07 (0.02, 0.11)**	0.06 (0.01, 0.11)*	0.03 (−0.02, 0.09)	0.03 (−0.03, 0.1)	0.03 (−0.05, 0.1)	−0.01 (−0.07, 0.06)
Fruit and Vegetable Preference						
Number of fruits rated *yucky*, *n* = 735	0.06 (−0.21, 0.32)	−0.05 (−0.39, 0.28)	−0.09 (−0.44, 0.27)	0.15 (−0.3, 0.59)	0.03 (−0.45, 0.52)	−0.11 (−0.54, 0.31)
Number of fruits rated *ok*, *n* = 735	−0.28 (−0.58, 0.02)	−0.01 (−0.38, 0.37)	0.03 (−0.38, 0.43)	−0.3 (−0.81, 0.2)	−0.03 (−0.59, 0.52)	0.27 (−0.21, 0.75)
Number of fruits rated *yummy*, *n* = 735	0.43 (0.12, 0.74)*	0.32 (−0.07, 0.71)	0.17 (−0.25, 0.59)	0.26 (−0.27, 0.78)	0.15 (−0.43, 0.72)	−0.11 (−0.61, 0.39)
Number of vegetables rated *yucky*, *n* = 735	0.25 (−0.11, 0.62)	0.15 (−0.31, 0.6)	0.01 (−0.47, 0.5)	0.24 (−0.37, 0.84)	0.13 (−0.53, 0.8)	−0.11 (−0.69, 0.47)
Number of vegetables rated *ok*, *n* = 735	−0.29 (−0.65, 0.07)	0.06 (−0.39, 0.51)	0.12 (−0.36, 0.6)	−0.41 (−1.01, 0.19)	−0.06 (−0.72, 0.59)	0.35 (−0.23, 0.92)
Number of vegetables rated *yummy*, *n* = 735	0.3 (−0.09, 0.69)	0.12 (−0.37, 0.6)	0.17 (−0.35, 0.69)	0.13 (−0.52, 0.77)	−0.05 (−0.76, 0.65)	−0.18 (−0.8, 0.44)
Gross Motor Skill						
Gross motor quotient^3^, *n* = 237	−2.42 (−6.4, 1.56)	−2.51 (−6.83, 1.81)	−8.1 (−12.26, −3.93)**	5.67 (−0.09, 11.44)	5.59 (−0.4, 11.58)	−0.08 (−5.95, 5.79)
Object control^3^, *n* = 245	−0.41 (−1.06, 0.24)	−0.1 (−0.83, 0.63)	−0.42 (−1.12, 0.28)	0.01 (−0.95, 0.97)	0.32 (−0.69, 1.34)	0.31 (−0.67, 1.29)
Locomotor^3^, *n* = 237	−0.36 (−1.39, 0.67)	−0.71 (−1.83, 0.4)	−2.25 (−3.32, −1.18)**	1.89 (0.41, 3.38)*	1.54 (−0.01, 3.08)	−0.35 (−1.87, 1.16)
Physical Activity						
MVPA, *n* = 685	16.77 (12.65, 20.88)**	8.75 (3.58, 13.93)**	11.16 (5.81, 16.51)**	5.6 (−1.15, 12.35)	−2.41 (−9.85, 5.04)	−8.01 (−14.62, −1.4)*
LPA, *n* = 685	0.94 (−5.84, 7.73)	6.72 (−1.84, 15.27)	5.02 (−3.8, 13.84)	−4.08 (−15.21, 7.05)	1.69 (−10.59, 13.98)	5.77 (−5.14, 16.69)
Sed/S, *n* = 685	−17.66 (−26.18, −9.14)**	−15.34 (−26.08, −4.6)*	−16.07 (−27.14, −4.99)**	−1.59 (−15.57, 12.38)	0.73 (−14.7, 16.15)	2.32 (−11.38, 16.02)
BMIz, *n* = 812	−0.04 (−0.08, 0.01)	−0.07 (−0.12, −0.01)*	0.03 (−0.03, 0.09)	−0.07 (−0.14, 0.01)	−0.09 (−0.18, −0.01)*	−0.03 (−0.1, 0.05)

*∆* Change, *CI* Confidence interval, *MVPA* Moderate to vigorous physical activity, *LPA* Light physical activity, *Sed/S* Sedentary time/sleep, *BMIz* Body mass index z-score

^1^ Results from linear mixed models adjusted for child age and race and ethnicity. Estimated values represent adjusted mean within group difference from baseline to endline. * *p* < 0.05; ** *p* < 0.01

^2^ Results from pairwise comparisons between study groups. Values represent mean between group differences in the change in score from baseline to endline. * *p* < 0.05; ** *p* < 0.01

^3^ 2017–2018 cohort only

**Table 3:** Moderation of intervention effects on nutrition-sensitive (willingness-to-try-new-foods) and physical activity-sensitive (moderate to vigorous physical activity) child outcomes by childcare center nutrition and physical activity environment quality in the CHAMP cluster-randomized controlled trial^1^

	Willingness-to-try-new-foods			Moderate to Vigorous Physical Activity	
	CHAMP vs. Control	CHAMP+ vs. Control		CHAMP vs. Control	CHAMP+ vs. Control
	Adjusted Estimate (95% CI)	Adjusted Estimate (95% CI)		Adjusted Estimate (95% CI)	Adjusted Estimate (95% CI)
**Center Nutrition Environment Quality**			**Center PA Environment Quality**		
Low	−0.05 (−0.15, 0.05)	−0.02 (−0.11, 0.07)	Low	−7.58 (−17.95, 2.78)	1.41 (−9.24, 12.07)
Moderate	0.04 (−0.03, 0.12)	0.05 (−0.03, 0.12)	Moderate	−0.46 (−8.18, 7.25)	7.88 (0.59, 15.16)*
High	0.14 (0.02, 0.26)*	0.11 (0, 0.22)*	High	6.65 (−6.22, 19.53)	14.34 (2.14, 26.55)*

*CI* Confidence interval

^1^Results are from pairwise comparisons of marginalized estimates of intervention effects at low (−1 SD), moderate (mean), and high (+ 1 SD) childcare center nutrition environment quality (nutrition Environment and Policy Assessment and Observation [EPAO]) and physical activity environment quality (physical activity Environment and Policy Assessment and Observation [EPAO]) between each intervention group and Control. Marginalized estimates are from linear mixed models adjusted for child age and race and ethnicity with a 3-way interaction between *intervention X time X childcare center EPAO score* (interaction p for each model: 0.06 for Willingness-to-try-new-foods and 0.27 for moderate to vigorous physical activity). Values represent mean between group differences in the change in child outcome from baseline to endline

* *p* < 0.05

** *p* < 0.01

#### Willingness-to-try-new-foods

Willingness-to-try-new-foods significantly increased in CHAMP and CHAMP+ and increased in Control over time with no difference among arms.

#### Fruit and vegetable preference

There was a small, significant increase in the number of fruits rated *yummy* in CHAMP from baseline to endline. There were no other meaningful changes in fruit and vegetable preferences within or between arms.

#### Gross motor skill

We restricted our analysis of gross motor competence to the 2017-2018 cohort (n = 22 centers, 245 children) after observing a significant association between data collector and the gross motor measures in the 2018-2019 cohort that aligned with staffing changes and data collection refresher training (Supplemental Table 7). Locomotor and gross motor quotient scores declined significantly in the Control arm, with slight declines in gross motor, object control, and locomotor scores in the intervention arms, such that at endline, locomotor scores were significantly higher in CHAMP (1.89 [95% CI: 0.41, 3.38]) and higher in CHAMP+ (1.54 [95% CI: −0.01, 3.08]) compared to Control. When pooled, locomotor scores and gross motor quotient were significantly higher in CHAMP/CHAMP+ compared to Control (locomotor CHAMP/CHAMP+ vs Control: 1.74 [95% CI: 0.43, 3.05]; gross motor quotient CHAMP/CHAMP+ vs Control: 5.67 [95% CI: 0.6, 10.75]).

#### Physical activity

MVPA significantly increased and sedentary time/sleep significantly decreased from baseline to endline in each arm. MVPA increased 5.60 (95% CI: −1.15, 12.35) mins/day in CHAMP compared to Control, not a statistically significant difference. MVPA increases were smallest in CHAMP+, such that MVPA increased significantly less in CHAMP+ compared to CHAMP (−8.01 mins/day [95% CI: −14.62,−1.40]).

#### BMIz

BMIz increased slightly in the Control arm. BMIz declined in CHAMP (−0.07 [95% CI: −0.14, 0.01]) and significantly declined in CHAMP+ (−0.09 [95% CI: −0.18, −0.01]) compared to Control. When pooled, we found a significant decline in CHAMP/CHAMP+ (−0.05 [95% CI: −0.08, −0.01]) and a significant difference from Control (−0.08 [95% CI: −0.15, 0]).

### Moderation by childcare center environmental quality

#### Nutrition-sensitive outcomes

**Fig. 2:**
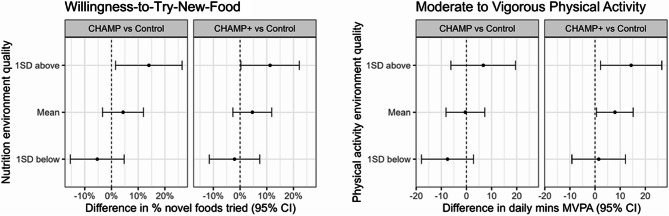
Moderation of intervention effects on nutrition- and physical activity-sensitive child outcomes by childcare center nutrition and physical activity environment quality in the CHAMP cluster-randomized controlled trial^1^ ^1^Results are from pairwise comparisons of marginalized estimates of intervention effects at low (−1 SD), moderate (mean), and high (+1 SD) childcare center nutrition environment quality (nutrition Environment and Policy Assessment and Observation [EPAO]) and physical activity environment quality (physical activity Environment and Policy Assessment and Observation [EPAO]) between each intervention group and Control. Marginalized estimates are from linear mixed models adjusted for child age and race and ethnicity with a 3-way interaction between *intervention X time X childcare center EPAO score*. Values represent mean between group differences in the change in child outcome from baseline to endline. p for interaction = 0.06 for willingness-to-try-new-foods and 0.27 for moderate to vigorous physical activity

Willingness-to-try-new-foods increased more in centers with higher quality nutrition environments (3-way interaction p = 0.06), with significant differences from Control for CHAMP and CHAMP+ (Table 3, Fig. 2).

#### Physical activity-sensitive outcomes

MVPA tended to increase more in centers with higher quality physical activity environments, with no statistically significant difference (Table 3, Fig. 2). In detail, there were significant differences in MVPA change between Control and CHAMP+ for children in moderate and higher quality environments, but not in lower quality environments.

## Discussion

This multi-component (diet and physical activity), multi-level (childcare and childcare + parent) trial among 56 childcare centers examined changes in children’s willingness-to-try-new-foods, fruit and vegetable preference, motor competence, physical activity, and weight gain. In addition, we examined if the effects were stronger when the intervention was extended to parents through an educational website, and if the impact of the intervention varied by centers' nutrition/physical activity environmental quality. 

### Nutrition outcomes 

We selected willingness-to-try-new-foods and fruit and vegetable preferences as nutrition-sensitive outcomes because they have been shown to be influenced by dietary interventions that can be implemented in childcare settings, including taste tests and observations of peers and staff eating new foods [26, 28, 57]. Studying children’s dietary choices in childcare without parents present provides insight on drivers that influence children’s dietary decisions [58]. Our findings illustrated that although children in the two intervention arms tried significantly more novel foods over the study period, the increases were modest, with no difference from Control. Likewise, although the CHAMP arm reported a significant increase in the number of foods rated as yummy, there were no differences in fruit and vegetable preference from Control. One explanation for the lack of intervention effects in either willingness-to-try-new-foods or fruit and vegetable preferences may be that children’s food hesitancies tend to abate over time, with increasing experiences with different foods and contexts [59]. Another explanation may be that taste preferences in young children do not necessarily transfer across modalities, such as tasting and rating a picture [60]. A third explanation may be that more intensive and engaging interventions, such as growing or preparing food, are needed to increase children’s willingness-to-try-new-foods and fruit and vegetable preferences, as suggested in a review of interventions [61]. A fourth explanation may be that individual differences in children’s appetitive traits and enjoyment could alter the impact of the intervention, as has been shown [58]. A final potential explanation is that the impact of the intervention may vary by the nutritional quality of the childcare setting, including staff modeling food-related behavior and practices [61].

 To examine the nutritional quality of the childcare centers, we used the EPAO ratings on food and beverages served, staff nutrition behavior, staff nutrition training and education, and nutrition policies [62]. The finding that in high nutrition quality centers, children in both intervention arms experienced significant increases in willingness-to-try-new-foods compared to Control supports the importance of considering the quality of the nutrition environment. Consistent with the bio-ecological model [11], children’s behavior is influenced directly and indirectly by their proximal interactions and experiences at home and in childcare. For example, childcare center practices such as serving fruits and vegetables first, followed by family style rather than plated meals has been related to increased fruit and vegetable consumption [63]. Practices, including staff eating with children, healthy food choices, responsive feeding, and avoidance of controlling and indulgent practices have also been associated with children’s eating behavior and consumption of healthy food [61]. Staff feeding practices may also relate to children’s eating behavior. In an analysis of baseline data, we found that children exposed to more indulgent childcare staff feeding practices had greater odds of demonstrating high willingness-to-try-new-foods, compared to children with less frequent exposure to indulgence [35]. 

### Physical activity outcomes 

We selected the Food Friends^®^ program because it includes a major focus on motor competence, which is a critical component of preschoolers’ development and influences their fitness, weight gain, and sports’ skills [43, 64, 65]. As Mighty Moves was implemented in the Fall, one consideration is that the Spring endline was conducted 12 weeks after the Mighty Moves intervention ended, potentially suggesting a maintenance effect, perhaps with fadeout, rather than an intervention effect [66]. Other possible explanations include declining staff enthusiasm or preschoolers reaching a plateau whereby they practiced emerging skills, rather than acquiring new skills. In contrast, the significant decline in the Control’s motor competence scores may suggest that gross motor development faltered without engagement in the intervention activities. This interpretation is supported by the finding that locomotor scores increased significantly in both intervention arms, and the difference with Control was significant in the CHAMP arm. 

 Motor competence and physical activity are closely aligned, such that they are thought to stimulate one another [67], illustrating why motor competence in this age group may be the target of interventions to promote physical activity and to prevent obesity. At baseline 75% of the children in this study attained the recommended 60 min of daily MVPA and MVPA was significantly associated with two motor competence components [68]. The increases in MVPA and decreases in sedentary time/sleep across all arms may relate to children’s increasing age. The higher increases in CHAMP, compared to CHAMP+ may be related to the physical space, as CHAMP+ centers were more frequently in urban rather than suburban sites with younger children. The intervention may not have been able to overcome the limited space and potentially limited equipment that often restricts children’s physical activity in childcare settings [69].

 As an exploratory hypothesis, we examined whether the impact of the intervention on children’s MVPA varied by the quality of the physical activity environment in the childcare settings. Quality, defined by the EPAO, included the setting (inside and outside), the time devoted to physical activity, the active and sedentary behavior of the children and staff, the staffs’ physical activity training and education, and physical activity policies. Our finding that in centers rated as moderate or high quality, time in MVPA was greater in CHAMP+ compared to Control centers is consistent with previous findings that context, including space, equipment, and the quality and quality of daily programming, can influence children’s physical activity [69]. 

### BMIz

The increase in BMIz in the Control is consistent with the increasing prevalence of weight gain among preschoolers nationally [3, 10]. In contrast, the decrease in BMIz in CHAMP and CHAMP+ suggests that the intervention may have contributed to a decrease in weight gain. In recent reviews of preschool interventions, many interventions have not shown an impact on anthropometry [70, 71]. One explanation may be that the weight gain in the Control arm children may be related to the lack of engagement in the dietary and physical activity behaviors promoted by the intervention. Another explanation may be that unmeasured aspects of the intervention positively impacted the children’s rate of weight gain, such that their BMIz scores declined. For example, although we do not have data on children’s dietary behavior outside of the childcare setting, taste tests may have increased children’s acceptance of novel healthier foods at home, contributing to a healthier weight gain and a decline in BMIz.

### Parent intervention

Our hypothesis that effects of the intervention would be strengthened by extending the intervention to parents through an educational website was not supported. The parent website was designed in consultation with parents with the goal of increasing parents’ access to the CHAMP curriculum and engaging parents in nutrition activities that children were experiencing in childcare. However, when it was operationalized, parent engagement was variable. In a separate investigation of the parent website, [29] we found that parents in each childcare center logged in a median of 15 times (IQR = 5-50; min-max = 1-112) for a median of 107.7 seconds (IRQ = 56.2-176.2; min-max = 0.0-256.0) throughout the intervention. In response to CHAMP Challenges where parents could respond, parents in each childcare center completed intervention activities a median of 2.5 times (IQR = 0-12.5; 0-20) [29]. 

 There were demographic differences in parent engagement [29]. Centers with a high proportion of parents who identified as other than non-Hispanic White and had less than a bachelor’s degree had significantly fewer webpage views and completed significantly fewer intervention activities compared to centers with parents who were predominantly non-Hispanic White and had more than a bachelor’s degree [29]. Parents noted that limited connectivity, time, and competing activities interfered with their participation. Another possibility is that working parents of preschoolers, who are often busy, may not have viewed their children’s diet or physical activity as a problem that needed to be addressed. Most children enrolled in the CHAMP trial (74%) had a BMIz within normal weight and the intervention may have been viewed as a center activity, rather than a home activity. Investigations of web-based interventions and mobile apps for parents have expressed concerns related to technology requirements, resulting in demographic and educational disparities in participation [72, 73]. Several reviews have shown little to no additional benefit of parent components combined with diet and physical activity interventions [69, 74]. These findings are concerning because children in low-income families are at increased risk for poor diet quality, including low fruit and vegetable intake [29, 75, 76] thus potentially widening health inequities. Subsequent plans include examining changes in parents’ behavior using implementation data in per-protocol analysis, including treatment-on-the-treated. These analyses can shed light on the components of the multi-component intervention that were or were not effective in altering parent behaviors. Future programs are needed that involve parents in the design and implementation of communication through web-based intervention, mobile phone apps, or other technology that is accessible to parents. 

### Strengths and weaknesses

There are both methodological strengths and weaknesses of this investigation. First, obtaining outcome measures directly from children using validated measures avoids the potential lack of awareness and bias associated with reports from caregivers and childcare staff. A second strength is the racially and ethnically diverse childcare centers across 10 geographically diverse counties. There are also several methodological challenges beyond the early termination of the trial due to the COVID-19 pandemic. Despite randomization, the CHAMP+ arm differed from the CHAMP and Control arms in locale (urban rather than suburban), racial mixture (high prevalence of non-Hispanic Black children), and age (younger children). In addition, loss-to-follow-up was higher in the CHAMP+ group. Demographic differences within the CHAMP+ arm were associated with parents’ engagement with the educational website and unadjusted demographic differences between arms may have biased the effect of the intervention on children’s dietary, motor competence, and physical activity. Although we controlled for these demographic variables in the analyses, we cannot rule out the possibility of selection bias. Children’s dietary behavior, physical activity, and weight are influenced by multiple household factors, including food insecurity, family resources, and caregiver-child engagement in dietary and physical activity behaviors [72, 73, 77], which were not measured or adjusted for in the analyses. The investigation of whether the impact of the intervention varied by the nutrition and physical activity environmental quality of the childcare environment was exploratory, highlighting the need for replication with a larger sample size. 

## Conclusions

To promote healthy growth and prevent excess weight gain among preschoolers, national policies and programs and the Dietary Guidelines of America have implemented strategies that focus on healthy dietary patterns. To promote physical activity, the World Health Organization developed guidelines for children under five years of age. These policies and recommendations have led to innovative programs that have increased the awareness of preschoolers’ need for healthful eating and physical activity [78]. However, the prevalence of obesity among preschoolers has continued to increase among children under age 5 years with current prevalence rates of 12.9% in the U.S. [79] and 8.5% globally [80]. 

 One explanation for the gap between health promoting policies and increases in obesity may be the lack of clarity and implementation guidance and support, as found in a review of movement policies in childcare settings [81]. Low quality nutrition and physical activity environments can dampen the impact of intervention programs on children’s dietary and physical activity behaviors, as shown in our findings. One possibility may be that in lower quality childcare centers, staff were not adherent to or engaged in the intervention. A recent review found that even when implementation policies to support healthy eating, physical activity, and obesity prevention were in place in childcare centers, there was very little impact on children’s diet, physical activity, or weight status [82, 83]. Future analyses are planned to examine differences in staff behavior based on the nutrition and physical activity quality of the centers. 

 Children are likely to need interactive programs with modeling and exploration in the context of high quality nutrition and physical activity environments to alter their dietary and physical activity behavior [83]. When implemented in Colorado, the Food Friends^®^ intervention showed longitudinal impacts on children’s willingness-to-try-new-food and motor competence, potentially because it was embedded in high quality nutrition and physical activity environments that included policy and systems support [26, 27, 83]. Our finding that the impact of the Food Friends^®^ intervention on willingness-to-try-new-foods and physical activity varied by the nutrition and physical activity environmental quality highlight the importance of focusing on an interactive program together with a high quality nutrition and physical activity environmental context. Thus, multi-level interventions that address the policy, systems, and environmental aspects of the setting, together with interactive, engaging programs for children may be necessary to alter children’s dietary and physical activity behavior and to sustain the effects. 

 Dietary and physical activity habits develop in early childhood and are often sustained throughout life, emphasizing a critical role for childcare centers [20, 84]. However, to date, few multi-level obesity prevention interventions in childcare centers have been implemented, and fewer have produced changes in adiposity [71]. As a consequence, little is known about the mechanisms driving childcare staff feeding and physical activity practices, staff adherence to interventions, and potential links to children's eating and physical activity behavior. In addition to supportive policies, systems, and environments, children benefit from high quality nutrition and physical activity environments [84], along with programs, such as the Food Friends^®^ curriculum, that provide frequent and engaging opportunities to practice and model healthy dietary and physical activity behaviors and to build healthy habits. These findings point to the need for research with multi-component (diet and physical activity) and multi-level interventions that include high quality nutrition and physical activity environments [84, 85]. Ensuring that childcare center policies and systems support high quality nutrition and physical activity environments and interactive intervention programs is critical to helping children build healthful dietary and physical activity habits that will reduce the risk of obesity and long-term chronic illnesses.

## Supplementary Information

Supplementary Material 1.

Supplementary Material 2.

Supplementary Material 3.

Supplementary Material 4.

## Data Availability

The dataset used during the current study is available from the corresponding author upon reasonable request.

## Abbreviations

BMIz: Body Mass Index Z-Score
CHAMP: Creating Healthy Habits Among Maryland Preschoolers
COVID-19: Coronavirus Disease-19
EPAO: Environment and Policy Assessment and Observation
FPL: Federal Poverty Level
MVPA: Moderate to Vigorous Physical Activity
SD: Standard Deviation
TGMD-2: Test of Gross Motor Development-2
