# Supplementary material for: Creating healthy habits for Maryland preschoolers (CHAMP): a cluster-randomized controlled trial among childcare centers
Source: Int J Behav Nutr Phys Act. 2025 Dec 10;22:156. doi: 10.1186/s12966-025-01824-6 (PMC12701592; doi:10.1186/s12966-025-01824-6)

Meet the Food Friends® (Character Introductions)

Character(s)	Mighty Moves® and Superpowers
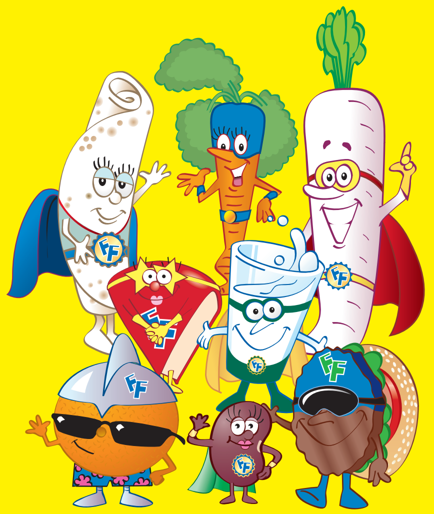	**The Food Friends®: Get Movin' With Mighty Moves™**<br>Meet the *Food Friends*: Corinne Carrot, Gertie Gouda, Ollie Orange, Marty Milk, Howie Hamburger, Tina Tortilla, and Wally Water Chestnut. Together with Bella Bean, they all love to move and have special skills. The *Food Friends* call these skills *Mighty Moves*. When the *Food Friends* use their *Mighty Moves*, they gain superpowers that make them healthy and strong.<br>The *Food Friends' Mighty Moves* take them on journeys throughout the town of Healthadelphia™. In Healthadelphia, they use their *Mighty Moves* to help others in their community. We are going to go on many journeys with the *Food Friends* and practice our own *Mighty Moves* so we can gain superpowers like them.
**Bella Bean™**<br>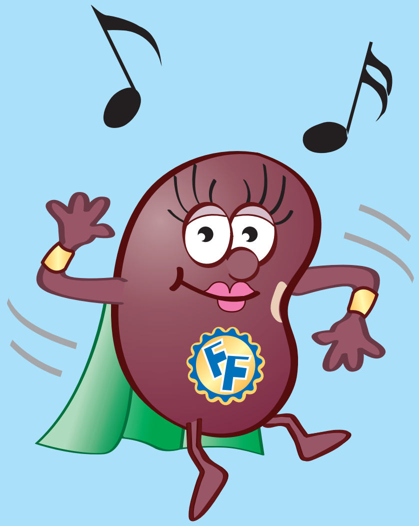	**Bella's Personality**<br>Bella is the mayor of Healthadelphia and is friendly and confident.<br>**Bella's Mighty Moves**<br>• Bella has great rhythm and loves to dance. Dancing to different kinds of music is her favorite thing to do.<br>• Bella knows body parts. She can move her arms, legs, and hips in all different ways.<br>• She is also the queen of space. Bella gives herself and those around her space to move.<br>**Bella's Superpower: Mind Reading**<br>Bella Bean always knows why you're smiling—or why you're sad. You never need to explain why you're crying or laughing to Bella because she has the amazing power to understand what you're thinking and what you're feeling. Bella's always the first to give you a hug when you're having a bad day because she'll know about it before you even say a word. She's also the first friend to give you a snack because she'll know you're hungry even before you do!

Background

Program Goal

The goal of *Mighty Moves*® is to enhance preschool children's gross motor skill development, in an effort to develop confidence in movement and increased physical activity levels. Further, *Mighty Moves* engages teachers by increasing awareness of the importance of gross motor skill development during early childhood and how skill development provides the foundation for healthy physical activity habits. The classroom can provide children with experiences to enhance motor skill development and physical activity levels.

Program Objectives

- To encourage children to develop gross motor skills, including locomotor, stability, and object manipulation skills
- To instill movement concepts, including body awareness, space awareness, action awareness, and movement-based learning
- To encourage and promote dramatic play and imagination
- To build community awareness
- To encourage the development of a positive attitude toward movement and physical activity
- To build and reinforce school-readiness skills

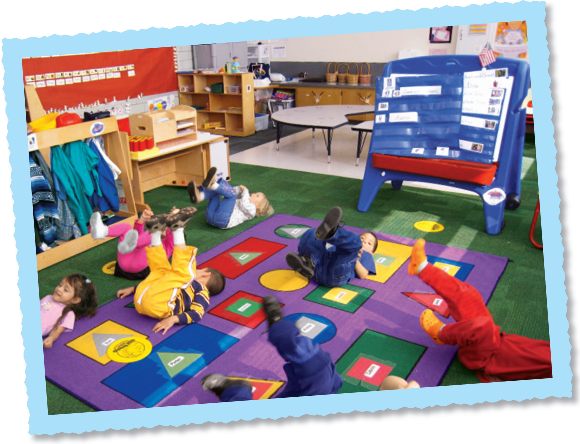

What is it?

The *Mighty Moves* program is comprised of two components: Teacher Training, Classroom Implementation. Both omponents are integral to the success of the program and work synergistically to establish a positive environment for children to practice and develop gross motor skills and to be active.

Who is it for?

Mighty Moves is developmentally-appropriate for three- to five-year-old children. It must be noted that there are major developmental differences between three-, four-, and five-year-olds. Therefore, throughout this program, you will see that the activities are designed with each age group and skill ability in mind.

Does it work?

Yes! In 2006, a *Mighty Moves* study was conducted in eight Head Start centers throughout Colorado. Physical fitness, gross motor skills, and physical activity were measured before and after program implementation. Data showed that children exposed to the program significantly increased gross motor skills and physical fitness when compared to the control group. Differences in motor ability, fitness, and physical activity were not seen among ethnic groups or by gender.

Why is it designed the way it is?

Creatively

Three- to five-year-olds learn through curiosity, exploration, and play. In order to appeal to their sense of fun and play, *The Food Friends*® characters were modified to include superpowers. The *Mighty Moves*® program uses Bella Bean™, Ollie Orange™, Tina Tortilla™, Marty Milk™, Howie Hamburger™, Corinne Carrot™, Gertie Gouda™, and Wally Water Chestnut to introduce gross motor skills and movement concepts to children. Children role-play these characters to classmates and teachers, thus reinforcing important health messages that the *Mighty Moves* program emphasizes. Whether using their superpowers or not, these characters create a positive, fun environment in which teachers interact with and model healthy behaviors to children.

Behaviorally

Recent research shows that children are not active enough and do not meet the national guidelines set for physical activity. Reasons for low activity levels among preschoolers are not well understood; however, there may be a relationship between children's motor skill performance and levels of physical activity. Several studies have shown that children with poor motor skills are less active than children with better-developed motor skills. Enhancing motor development at this age is critical. Children who never develop mature motor patterns in early childhood will continue to perform poorly as they grow older. Children with poor motor development feel awkward, uncoordinated, and have less confidence; therefore, they move less. Efforts to increase motor skill ability in young children may help them increase their physical activity levels and enjoyment. *Mighty Moves* was designed to engage children in activities that promote motor skill development and encourage children to be physically active.

Developmentally

Humans are "wired" to move. Brain research demonstrates that each area of child development—physical, social/emotional, and cognitive—grows and matures together. Young children learn best by doing. They need to engage their whole bodies through hands-on experiences to fully activate the brain in the learning process. Movement experiences are optimal for brain development and have constant interplay. When we move, we stimulate the brain by creating synapses, which connect the cerebellum portion of the brain to cognitive functions such as memory, attention, spatial relationships, language, and other functions that are needed for successful learning.

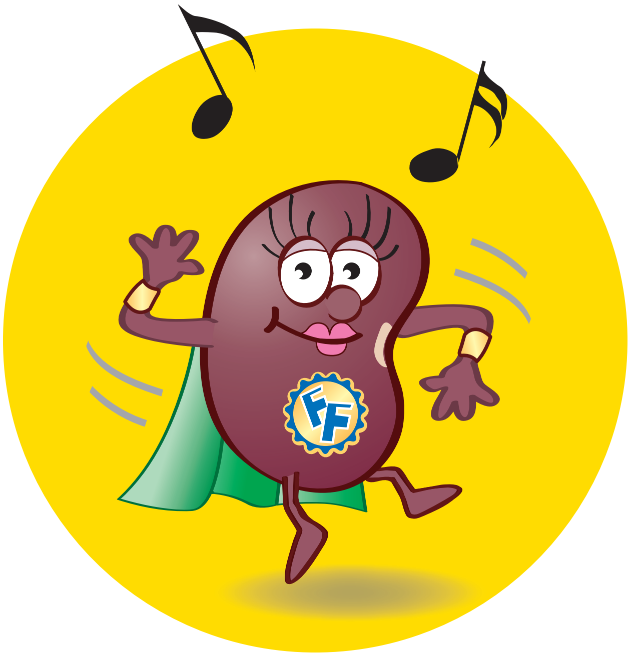

In the Classroom

The design of the *Mighty Moves*® program is based on input provided by those who the program intends to reach: preschoolers and teachers. Teachers participated in telephone interviews and group discussions, as well as in reviewing lesson plans. Preschoolers were asked about graphics and what types of activities they like to do. The result of those conversations is the *Mighty Moves* program.

The Food Friends®: Get Movin' With Mighty Moves™

- Superhero theme
- 18 weeks
- 4 (15–20 minute) lessons a week
- Music and equipment provided
- Focus on physical activity levels and gross motor skill development
- Incorporate school-readiness skills
- Reinforce social skills such as cooperation, kindness, empathy, and sharing
- Encourage and promote dramatic play and imagination
- Build community awareness
- Each of the 8 characters has a superpower, *Mighty Moves* (motor skills), music, and leads kids in activities for 2 weeks.

Healthadelphia™

Nestled in green rolling hills, Healthadelphia is the name of the town where the *Food Friends* live, play, and work. Amongst the trees, plants, and flowers, you will find gardens, a farm, a restaurant, a supermarket, a library, a zoo, parks, a laundromat, police and fire stations, and a post office.

The *Food Friends* will lead the children on musical journeys and imaginary trips to help the citizens of Healthadelphia. The musical journeys take the children to places where food is grown, sold, or eaten. These journeys are conducted to music and are repeated several times. Children will also take imaginary trips to other community establishments and learn about the jobs and tasks people do in these settings. Children will use movement narratives to explore community sites on the imaginary trips.

♬ *Musical Journeys to the:*

- Garden with Bella Bean™
- Farmers' Market with Corinne Carrot™
- Farm with Gertie Gouda™
- Restaurant with Ollie Orange™
- Supermarket with Marty Milk™
- Picnic with Howie Hamburger™
- Vineyard/Orchard with Tina Tortilla™
- Winter Carnival/County Fair with Wally Water Chestnut

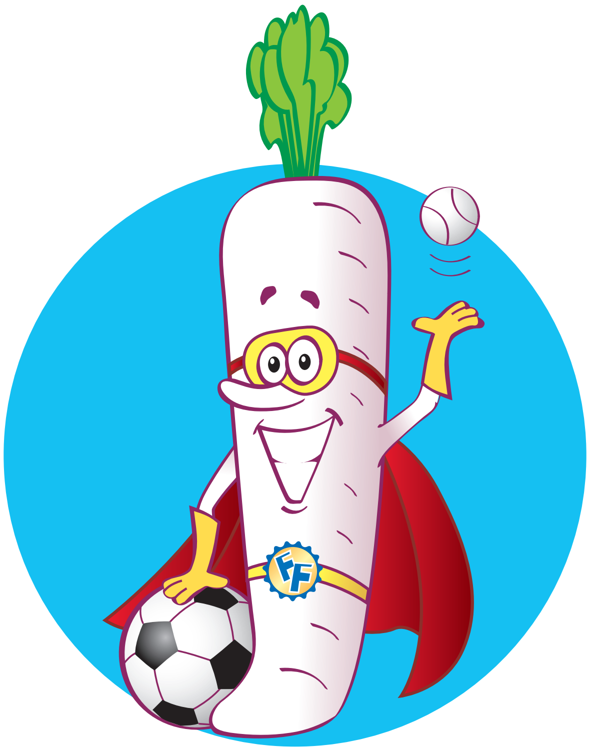

Program Overview

Imaginary Trips to community sites to help out:

- Lucy the Librarian at the Library
- Zelda the Zookeeper at the Zoo
- Miguel the Mechanic at the Auto Shop
- Lifeguard Luisa at the Beach
- Ranger Ricardo at the Park
- Mom and Dad at the Laundromat
- Tim the Ticket-taker at the Theater
- Patti Policewoman at the Police Station
- Cathy the Clown at the Circus
- Fireman Frank at the Firehouse
- Builder Bryan at the Building Site
- Mia the Mailwoman at the Post Office

Classroom Materials

Teacher Guide (1) – This will guide you through the *Mighty Moves*® program in your classroom. Lessons are outlined and learning objectives are identified.

Musical CD (1) –

Original music made specifically for *Mighty Moves* is provided on a 17-track CD. The CD includes a theme song, voice introductions of all eight characters, and eight musical songs for journeys.

Activity Mats (20) – Activity Mats are the cornerstone of the program, as they are used to teach kids about space, as targets, as boundaries, and as parts of obstacle courses.

Puppets (8) – The puppets will help you to teach Mighty Moves and the characters' superpowers.

Activity Cards (9) – Activity Cards outline each of the eight character's *Mighty Moves* and superpowers.

Beanbags (20) –Beanbags will be used to practice balancing, tossing, passing, and catching.

Balls (15) – Two different kinds of balls are included: Activity Balls (6) and Sensory Balls (9). Balls will be used in the latter half of the program for catching, throwing, and kicking.

Activity Scarves (Variable) – Activity Scarves are included to serve as capes, as well as to introduce object manipulation skills. Scarves are also used in the "Caping Ceremony" in Week 10.

Rope (1) – A rope is included to be used as a tightrope, to make pathways, and for obstacle courses.

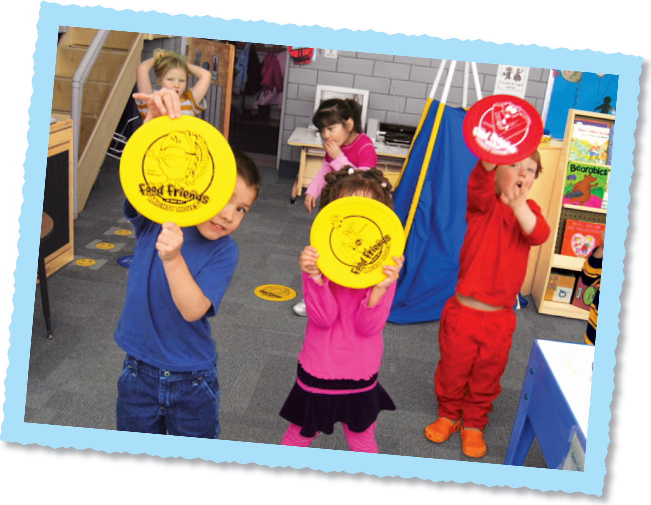

Lesson 1 Overview:

The Food Friends®: Get Movin' With Mighty Moves™

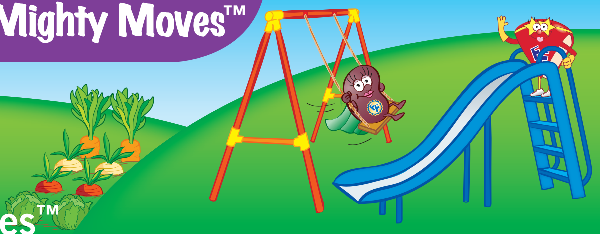

Lesson Description

Children will learn about *The Food Friends*® characters and *Mighty Moves*®. They will learn (or review) the safety rules that are expected when conducting *Mighty Moves*. They are: keep ourselves safe, keep each other safe, and keep our things safe.

Materials:

- ♫ *Mighty Moves* Musical CD (and CD player)
- *Food Friends* Puppets
- Activity Cards
- Activity Mats

Learning Objectives

Children will:

- be introduced to the *Food Friends* characters.
- be introduced to *Mighty Moves*.
- show progress in understanding and following simple and multiple-step directions.
- expand knowledge of and respect for their bodies and the environment.

Movement Concepts

- *Mighty Moves*

Lesson Plan

1. Read the activities on the next page and become familiar with the activities.
2. Conduct the following activities:

A. Introducing the Food Friends
- Show each of the *Food Friends* Puppets.

B. Mighty Moves Safety Rules
- Explain the Safety Rules.

C. Introducing Activity Mats
- Show the Activity Mats and explain their purpose.
- Scatter the Activity Mats throughout the room and have each child stand on one.

D. Introducing Mighty Moves
- Use the front of the Activity Card, Puppet, and the CD to introduce each character.

E. ♫ Mighty Moves Theme Song
- Play Track 1 and lead the children in acting out the movements.

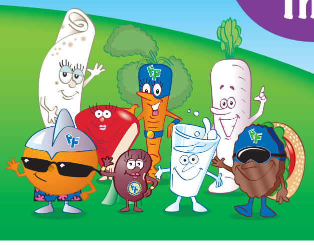

Lesson 1: The Food Friends®: Get Movin' With Mighty Moves™

Activities

A. Introducing the Food Friends®

Today we have visitors. The *Food Friends* characters are here to teach us how to be healthy. We are going to do lots of fun and new *Mighty Moves®* with the *Food Friends* every day. Are you ready to meet the *Food Friends*!? Here they are!

B. Mighty Moves Rules

Before we start our *Mighty Moves,* we need to talk about our safety rules.
When we do *Mighty Moves,* we always need to:

- keep ourselves safe.
- keep each other safe.
- keep our things safe.

C. Introducing Activity Mats

These are our Activity Mats and they will help us keep our own space and follow the safety rules.

D. Introducing Mighty Moves

Now let's learn about our new *Food Friends' Mighty Moves*.

- *Bella Bean™* – Loves to Dance *(Track 2)*. Can you dance on your mat like Bella?
- *Corinne Carrot™* – Twists and Turns *(Track 4)*. Can you twist and turn on your mat like Corinne?
- *Gertie Gouda™* – Walks, Marches, and Gallops *(Track 6)*. Can you march on your mat like Gertie?
- *Ollie Orange™* – Skates, Hops, and Slides *(Track 8)*. Can you skate on your mat like Ollie?
- *Marty Milk™* – Loves to Be Strong *(Track 10)*. Can you be strong like Marty?
- *Howie Hamburger™* – Bikes, Swims, and plays outdoors *(Track 12)*. Can you pretend to bike like Howie?
- *Tina Tortilla™* – Jumps *(Track 14)*. Can you jump in place like Tina?
- *Wally Water Chestnut* – Kicks and Throws *(Track 16)*. Can you pretend to throw a ball? Can you kick a ball like Wally?

E. ♫ Mighty Moves Theme Song

Guess what? The *Food Friends* even have their own song about *Mighty Moves*. Raise your hand if you want to hear their song. We can dance to *The Food Friends* song. Everyone stand on your mat and get ready to move.

The Food Friends *say raise your hand if you like moving like the* Food Friends *with* Mighty Moves. *Great! We are going to practice different* Mighty Moves *each day.*

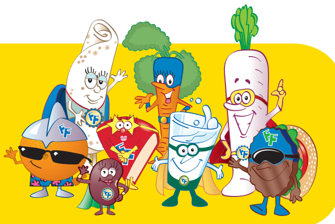

Supplement: Supplementary file 1 — Supplementary Material 1. [file 12966_2025_1824_MOESM1_ESM.pdf]
