# Supplementary material for: Creating healthy habits for Maryland preschoolers (CHAMP): a cluster-randomized controlled trial among childcare centers
Source: Int J Behav Nutr Phys Act. 2025 Dec 10;22:156. doi: 10.1186/s12966-025-01824-6 (PMC12701592; doi:10.1186/s12966-025-01824-6)
Supplement: Supplementary file 2 — Supplementary Material 2. [file 12966_2025_1824_MOESM2_ESM.pdf]

a custom solution from Lakeshore®

The Food Friends®

~ FUN WITH ~ NEW FOODS™

Teacher's Guide

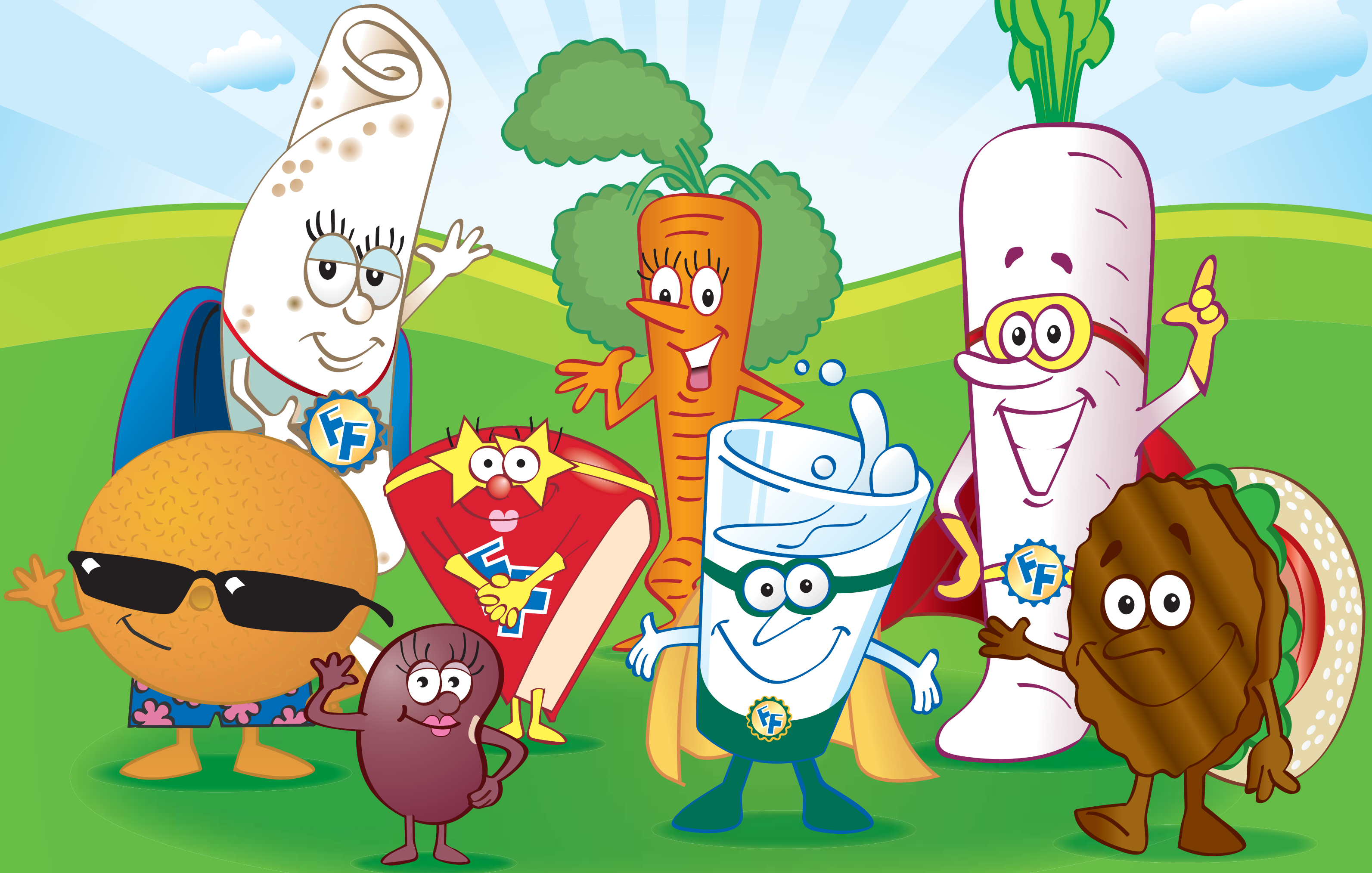

Establishing Healthy Eating Behaviors in Young Children

www.childcarechamp.org

©2009 Colorado State University

Program goal

The goal of *Fun With New Foods*™ is to increase children's willingness to try new foods in an effort to increase food choice and, hence, dietary variety. Further, *Fun With New Foods*™ reaches teachers by increasing their awareness of the importance of offering new foods and educating them on how to introduce new foods to preschoolers.

Program Objectives

- To encourage the children to develop a willingness to taste new foods
- To build familiarity with new foods
- To encourage the development of a positive attitude toward trying new foods
- To encourage and promote a positive feeding environment
- To instill basic nutritional concepts in the children
- To build school readiness skills, including:
  - Language development
  - Literacy
  - Mathematics
  - Science
  - Creative arts
  - Social and emotional development
  - Approaches to learning
  - Physical health and development

What Is It?

The *Fun With New Foods*™ program is comprised of two components: Teacher Training and Classroom Implementation. Both components are integral to the success of the program and work synergistically to establish a positive feeding environment for children to experience and try new foods.

Who Is It For?

Fun With New Foods™ is developmentally appropriate for three- to five-year-old children. Secondary influencers—teachers receive educational information.

Does It Change Behavior?

Yes! In 2000, a *Fun With New Foods*™ pilot study was conducted in four Head Start centers (two rural and two urban). Food preference panels of familiar and new foods were administered before and after program implementation, and observations of individual children's responses to new foods during program tasting opportunities were also included in the study. Data from our pilot food preference panel assessments showed that children exposed to the program significantly decreased the number of food refusals to new foods compared to children in a control group. This outcome is indicative of an increase in their willingness to try new foods, the first stage of food acceptance. Data from past years indicate that the program continues to work.

What Is a New Food?

For the *Fun With New Foods*™ classroom component, a new food has been defined as a food that at least 85% of children have tried four or fewer times in his or her lifetime. The foods identified for tasting in the classroom are new to the majority of preschoolers in Maryland. They are used in the program as a mechanism to change behavior in preschoolers in the classroom environment. Collectively, these foods are NOT intended to be representative or prescriptive of what preschoolers should be eating. However, they all fit into a healthy diet.

New foods at home may not be the same as the new foods offered in the classroom component. A new food at home can be any food that is new to the family or, specifically, to the child. This can be as simple as a different colored apple or a different shaped pasta. It can also be a new type of vegetable or bread, or a new way of preparing a food (think potatoes—scalloped, boiled, mashed, baked, fried, etc.). What is new to one family may be common to another. So, a new food is defined for families as something that is not part of their regular diet at the time.

For some families, it is important to consider the price of new foods. Many children will develop a preference and liking for water chestnuts. Children have been known to go home and ask for water chestnuts. It can be expensive and may not fit into every family's food budget.

It is important to note that the message to parents should not be to purchase specific foods but rather to bring their child to the new sections of the grocery store and have their child pick out a new kind of food that fits into the family budget.

Why Is It Designed the Way It Is?

Fun With New Foods™ is target audience driven. Preschoolers and teachers had, and still have, a tremendous amount of input into the program. The characters were chosen by kids; the kit (with the idea that all materials need to be in one place) was teacher-driven, as were many of the activities.

Creatively

Three- to five-year-olds learn through curiosity, exploration, and play. In order to appeal to their sense of fun and play, cartoon characters were created. The *Fun With New Foods*™ program uses familiar food characters—Bella Bean, Ollie Orange, Tina Tortilla, Marty Milk, Howie Hamburger, and Corrine Carrot—to introduce their "new friends" Gertie Gouda and Wally Water Chestnut to the class. Children role-play these characters to classmates, teachers, and parents, thus reinforcing important health messages that the *Fun With New Foods*™ program emphasizes. Whether using their superpowers or not, these characters create a positive, fun environment in which teachers interact with and model healthy behaviors to children.

Behaviorally

The preschool years are a time when eating habits are established, thus it is a perfect time for interventions aimed at healthful eating. Also, research and our own pilot project suggest that it can take up to eight to twelve exposures to a new food before a child is familiar enough with it to put it in his or her mouth and swallow it. As a result, the *Fun With New Foods*™ program is designed to be twelve weeks in length with multiple opportunities for tasting and learning about new foods.

So, Why Spend the Time and Resources on Preschoolers and Picky Eating?

Eating habits are established early in life—during the first six years. These habits are carried into adulthood. Adults who have a more varied diet have less risk for some chronic diseases, such as diabetes, cardiovascular disease, cancer, and obesity. It is hypothesized that if we can increase children's willingness to try new foods, enhance their dietary variety, and establish healthy eating habits, then they may have decreased risk for some chronic diseases as adults.

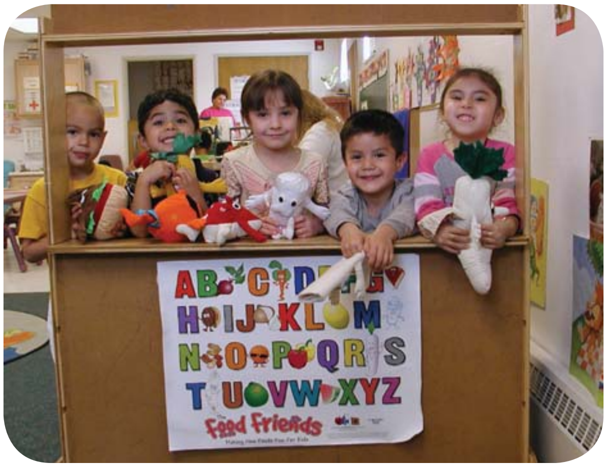

Introducing the Characters

The *Fun With New Foods*™ program was built upon the concept that familiarity with food leads to intake, and intake leads to preference. Thus, to pictorially depict this concept, eight characters were created—six representing foods familiar to preschoolers and two representing new foods. The characters represent all five major food groups and have distinct personalities.

Not only are *The Food Friends*® regular characters, but they are also superheroes who will teach the children superhero attributes, such as cooperation, sharing, empathy, kindness, intelligence, and problem-solving abilities. Children will learn that the superpowers of *The Food Friends*® are available when needed in order to promote a healthy lifestyle.

Drumroll, please…Introducing *The Food Friends*®! (Please note, superpowers are identified in italics.)

Familiar Friends (Foods)

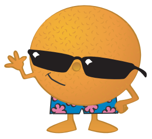

Ollie Orange
the *lightning-quick* cool dude

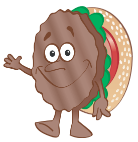

Howie Hamburger
the *transforming* outdoor enthusiast

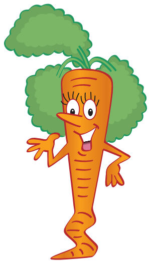

Corrine Carrot
the curious and inquisitive Food Friend with *X-ray vision*

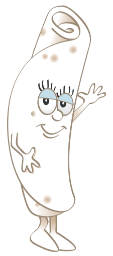

Tina Tortilla
the joyful Food Friend who can *fly*

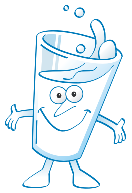

Marty Milk
the problem-solver with *super-strength*

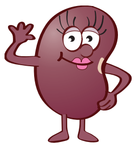

Bella Bean
the confident mayor of Healthadelphia who can *read minds*

New Friends (Foods)

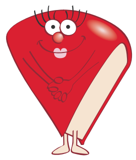

Gertie Gouda
the loving, *super-stretchable* Food Friend

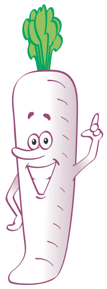

Wally Water Chestnut
the determined and focused Food Friend who can become *invisible*

Fun With New Foods™ Weekly Outline

Week	Character(s)	Day 1	Day 2
1	Everyone 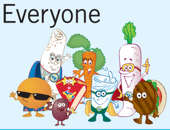	Activity: *The Food Friends*® Puppet Show<br>Food: Kidney Bean	Activity: *Fun With New Foods*™ Activity Cards<br>Food: Water Chestnut
2	Corrine 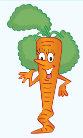	Activity: Read *I Will Never NOT EVER Eat a Tomato*<br>Food: Kidney Bean	Activity: *Fun With New Foods*™ Fruit and Vegetable<br>Food: Water Chestnut
3	Bella 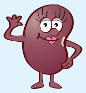	Activity: Read *Bread and Jam for Frances*<br>Food: Kidney Bean	Activity: *Fun With New Foods*™ Theme Song<br>Food: Water Chestnut
4	Wally 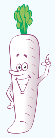	Activity: Matching Smells<br>Food: Kidney Bean	Activity: Talking About Favorite Smells<br>Food: Water Chestnut
5	Howie 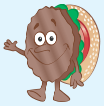	Activity: Read *Yoko*<br>Food: Kidney Bean	Activity: Art With Howie<br>Food: Water Chestnut
6	Gertie 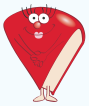	Activity: Read *Eating the Alphabet*<br>Food: Kidney Bean	Activity: *Fun With New Foods*™ from A to Z<br>Food: Water Chestnut
7	Ollie 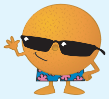	Activity: Couscous Sand Art<br>Food: Couscous	Activity: Couscous and Fruit Recipe<br>Food: Water Chestnut
8	Tina 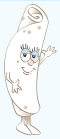	Activity: Play Dough Tortillas<br>Food: Kidney Bean	Activity: Food Collage<br>Food: Water Chestnut
9	Marty 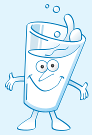	Activity: Read *Dragon's Love Tacos*<br>Food: Wasa Bread	Activity: *Fun With New Foods*™ Memory Cards<br>Food: Beets
10	Ollie, Bella, Corrine, Howie 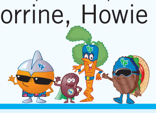	Activity: *Fun With New Foods*™ Activity Book<br>Food: Edamame	Activity: *The Food Friends*® Detectives<br>Food: Dried Currents
11	Tina, Gertie, Marty, Wally 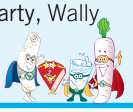	Activity: Read *Green Eggs and Ham*<br>Food: Lychee	Activity Rhyming With *The Food Friends*®<br>Food: Hearts of Palm
12	Everyone 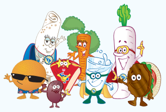	Activity: One of These Foods Is Not Like the Other<br>Food: Hominy	Activity: *Fun With New Foods*™ Tasting Party and Super Taster Ceremony<br>Food: Tasting Party Foods
