# Supplementary material for: Creating healthy habits for Maryland preschoolers (CHAMP): a cluster-randomized controlled trial among childcare centers
Source: Int J Behav Nutr Phys Act. 2025 Dec 10;22:156. doi: 10.1186/s12966-025-01824-6 (PMC12701592; doi:10.1186/s12966-025-01824-6)
Supplement: Supplementary file 3 — Supplementary Material 3. [file 12966_2025_1824_MOESM3_ESM.pdf]

Supplemental Appendix B: Examples of Content Published on the Parent Intervention Website

Parenting Infographics

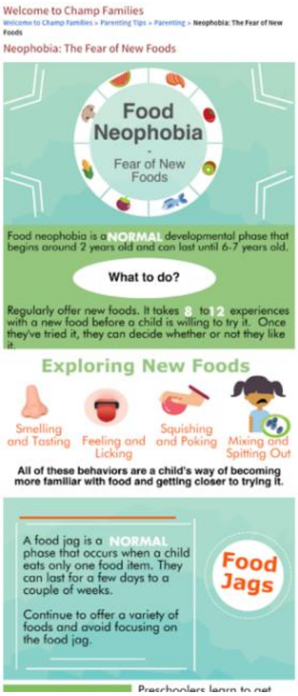

Recipes

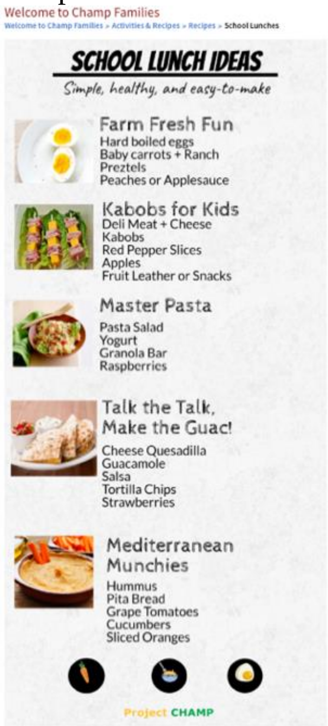

CHAMP Challenges

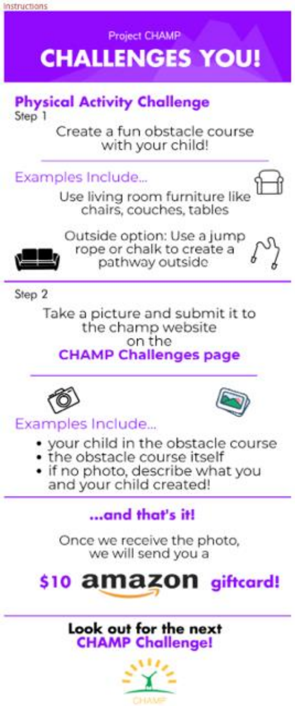

Videos

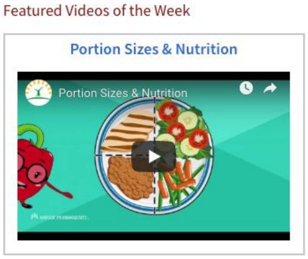

Quizzes

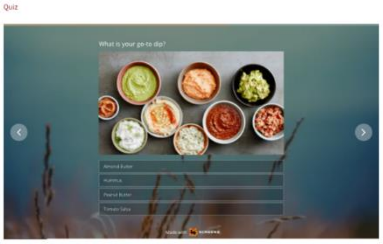

Photos of Child Participating in Intervention Activities at Childcare Center

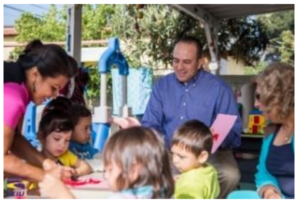

Supplemental Figure Caption

Intervention website content was updated each week and included parenting infographics, nutritious and child-friendly recipes, intervention activities (CHAMP Challenges), short videos on nutrition, quizzes on food and health information, and photos of children participating in the intervention-related activities at their childcare center.
