# Supplementary material for: Creating healthy habits for Maryland preschoolers (CHAMP): a cluster-randomized controlled trial among childcare centers
Source: Int J Behav Nutr Phys Act. 2025 Dec 10;22:156. doi: 10.1186/s12966-025-01824-6 (PMC12701592; doi:10.1186/s12966-025-01824-6)
Supplement: Supplementary file 4 — Supplementary Material 4. [file 12966_2025_1824_MOESM4_ESM.docx]

Supplemental Table 1. Baseline characteristics of childcare centers retained and lost to follow-up in the CHAMP cluster-randomized controlled trial^1^

|  | **Childcare centers retained** | **Childcare centers lost to follow-up** | **p^2^** |
| --- | --- | --- | --- |
| Center Characteristics | n = 49 | n = 7 |  |
| Study Group |  |  | 0.89 |
| CHAMP | 18 (37%) | 2 (29%) |  |
| CHAMP+ | 16 (33%) | 3 (43%) |  |
| Control | 15 (31%) | 2 (29%) |  |
| Mean child participants/center | 16.98 (9.82) | 9.86 (4.53) | 0.07 |
| Center size, ≤50 children | 35 (71%) | 4 (67%) | 1.00 |
| CACFP participation, n (%) | 21 (44%) | 3 (50%) | 1.00 |
| Children’s race and ethnicity |  |  |  |
| >70% White | 18 (37%) | 0 (0%) | 0.12 |
| >70% Black | 23 (47%) | 4 (67%) |  |
| No group >70% | 8 (16%) | 2 (33%) |  |
| Mean center income, % federal poverty line | 288.13 (99.92) | 220.05 (160.94) | 0.13 |
| Locale |  |  | 0.48 |
| Rural | 6 (12%) | 0 (0%) |  |
| Suburban | 28 (57%) | 6 (86%) |  |
| Urban | 15 (31%) | 1 (14%) |  |

Abbreviations: SD, standard deviation; CACFP, Child and Adult Care Food Program.

^1^ Presented as mean (SD) or n (%), as appropriate

^2^ P value from Student’s t-test or Fisher’s exact test. * p < 0.05; ** p < 0.01

Supplemental Table 2. Baseline characteristics of children retained or lost to follow-up in the CHAMP cluster-randomized controlled trial^1^

|  | **Children retained** | **Children lost to follow-up** | **p^2^** |
| --- | --- | --- | --- |
| Center Characteristics | n = 627 | n = 235 |  |
| Study Group |  |  | <0.01** |
| CHAMP | 286 (46%) | 80 (34%) |  |
| CHAMP+ | 182 (29%) | 97 (41%) |  |
| Control | 159 (25%) | 58 (25%) |  |
| Mean child participants/center | 22.03 (10.92) | 20.96 (12.49) | 0.22 |
| Center size, ≤50 children | 194 (67%) | 82 (77%) | 0.06 |
| CACFP participation, n (%) | 160 (57%) | 51 (52%) | 0.38 |
| Children’s race and ethnicity |  |  | <0.01** |
| >70% White | 98 (34%) | 6 (6%) |  |
| >70% Black | 129 (44%) | 65 (61%) |  |
| No group >70% | 63 (22%) | 35 (33%) |  |
| Mean center income, % federal poverty line | 315.17 (85.13) | 278.36 (115.86) | <0.01** |
| Locale |  |  | 0.15 |
| Rural | 98 (16%) | 36 (15%) |  |
| Suburban | 368 (59%) | 153 (65%) |  |
| Urban | 161 (26%) | 46 (20%) |  |
| Observed nutrition environment score  (EPAO range: 0-21) | 8.11 (1.38) | 7.43 (1.22) | <0.01** |
| Observed physical activity environment score  (EPAO range: 0-36) | 11.33 (2.01) | 10.67 (1.96) | <0.05* |
| Child Characteristics | n = 627 | n = 235 | p^1^ |
| Age, months | 48.39 (7.37) | 48.57 (7.87) | 0.76 |
| Gender, male | 338 (54%) | 131 (56%) | 0.69 |
| Race and ethnicity |  |  | <0.01** |
| non-Hispanic White | 353 (57%) | 84 (38%) |  |
| non-Hispanic Black | 188 (31%) | 104 (47%) |  |
| all other^3^ | 74 (12%) | 35 (16%) |  |
| Willingness-to-try-new-foods, % novel foods tried | 0.33 (0.35) | 0.34 (0.34) | 0.80 |
| Fruit and Vegetable Preference |  |  |  |
| Number of fruits rated *yucky* | 2.09 (1.9) | 2.14 (1.95) | 0.81 |
| Number of fruits rated *ok* | 1.73 (2.15) | 1.59 (2.17) | 0.48 |
| Number of fruits rated *yummy* | 4.95 (2.53) | 4.99 (2.61) | 0.87 |
| Number of vegetables rated *yucky* | 3.73 (2.97) | 4.05 (3.29) | 0.26 |
| Number of vegetables rated *ok* | 1.96 (2.58) | 1.52 (2.26) | 0.07 |
| Number of vegetables rated *yummy* | 4.98 (3.25) | 5 (3.29) | 0.95 |
| Gross Motor Skill |  |  |  |
| Gross motor^4^ | 123.24 (14.12) | 120.25 (16.65) | 0.24 |
| Locomotor^4^ | 14.16 (3.57) | 13.18 (3.4) | 0.11 |
| Object control^4^ | 13.59 (2.3) | 13.35 (3.26) | 0.56 |
| Physical Activity |  |  |  |
| MVPA, mins/d | 75.69 (24.28) | 78.45 (40.84) | 0.30 |
| LPA, mins/d | 369.75 (50.99) | 354.84 (45.08) | <0.01** |
| Sed/S, mins/d | 994.57 (62.91) | 1006.71 (65.85) | <0.05* |
| BMI-Z | 0.45 (1.05) | 0.36 (1.1) | 0.28 |

Abbreviations: SD, standard deviation; CACFP, Child and Adult Care Food Program; EPAO, Environment and Policy Assessment and Observation; MVPA, moderate to vigorous physical activity; LPA, light physical activity; Sed/S, sedentary time/sleep; BMIz, body mass index z-score.

^1^ Presented as mean (SD) or n (%), as appropriate

^2^ P value from Student’s t-test or Chi-square test. * p < 0.05; ** p < 0.01

^3^ Child all other race and ethnicity includes individuals from the following groups: non-Hispanic multiracial (n = 55); Hispanic of any race (n = 29); non-Hispanic other race (n=24); unknown race and ethnicity (n=1).

^4^ 2017-2018 cohort only

Supplemental Table 3. Baseline to endline changes in objective child measures within and between study arms in the CHAMP cluster-randomized controlled trial

|  | **CHAMP/CHAMP+** | **Control** | **CHAMP/CHAMP+ vs Control** |
| --- | --- | --- | --- |
|  | Mean ∆ (95% CI)^1^ | Mean ∆ (95% CI)^1^ | Adjusted Estimate (95% CI)^2^ |
| Willingness-to-try-new-foods, n = 757 | 0.06 (0.03, 0.09)** | 0.03 (-0.02, 0.09) | 0.03 (-0.03, 0.09) |
| Fruit and Vegetable Preference |  |  |  |
| Number of fruits rated *yucky*, n = 735 | 0.01 (-0.19, 0.22) | -0.09 (-0.44, 0.27) | 0.10 (-0.31, 0.51) |
| Number of fruits rated *ok*, n = 735 | -0.17 (-0.40, 0.07) | 0.03 (-0.38, 0.43) | -0.19 (-0.66, 0.27) |
| Number of fruits rated *yummy*, n = 735 | 0.39 (0.14, 0.63)** | 0.17 (-0.25, 0.59) | 0.21 (-0.27, 0.70) |
| Number of vegetables rated *yucky*, n = 735 | 0.21 (-0.07, 0.49) | 0.01 (-0.47, 0.50) | 0.2 (-0.37, 0.76) |
| Number of vegetables rated *ok*, n = 735 | -0.15 (-0.43, 0.13) | 0.12 (-0.36, 0.60) | -0.27 (-0.83, 0.29) |
| Number of vegetables rated *yummy*, n = 735 | 0.22 (-0.08, 0.53) | 0.17 (-0.34, 0.69) | 0.05 (-0.55, 0.65) |
| Gross Motor Skill |  |  |  |
| Gross motor quotient^3^, n = 237 | -2.43 (-5.34, 0.49) | -8.1 (-12.25, -3.94)** | 5.67 (0.60, 10.75)* |
| Object control^3^, n = 245 | -0.27 (-0.76, 0.22) | -0.42 (-1.12, 0.28) | 0.15 (-0.7, 1.00) |
| Locomotor^3^, n = 237 | -0.51 (-1.27, 0.24) | -2.25 (-3.32, -1.18)** | 1.74 (0.43, 3.05)* |
| Physical Activity^4^ |  |  |  |
| LPA, n = 685 | 3.17 (-2.14, 8.48) | 5.03 (-3.78, 13.84) | -1.86 (-12.15, 8.43) |
| Sed/S, n = 685 | -16.76 (-23.44, -10.09)** | -16.09 (-27.16, -5.02)** | -0.67 (-13.60, 12.25) |
| BMIz, n = 812 | -0.05 (-0.08, -0.01)* | 0.03 (-0.03, 0.09) | -0.08 (-0.15, 0.00)* |

Abbreviations: ∆, change; CI, confidence interval; MVPA, moderate to vigorous physical activity; LPA, light physical activity; Sed/S, sedentary time/sleep; BMIz, body mass index z-score.

^1^ Results from linear mixed models adjusted for child age and race and ethnicity. Intraclass correlation ranged from 0% for number of vegetables rated *ok* to 5.9% for LPA. Estimated values represent adjusted mean within group difference from baseline to endline. * p < 0.05; ** p < 0.01

^2^ Results from pairwise comparisons between study groups. Values represent mean between group differences in the change in score from baseline to endline. * p < 0.05; ** p < 0.01

^3^ 2017-2018 cohort only

^4^ Moderate to vigorous physical activity not included due to significant differences between CHAMP and CHAMP+ intervention arms.

Supplemental Table 4. Unadjusted baseline to endline changes in objective child measures within and between study arms in the CHAMP cluster-randomized controlled trial

|  | **CHAMP** | **CHAMP+** | **Control** |  | **CHAMP vs Control** | **CHAMP+ vs Control** | **CHAMP+ vs CHAMP** |
| --- | --- | --- | --- | --- | --- | --- | --- |
|  | Mean ∆ (95% CI)^1^ | Mean ∆ (95% CI)^1^ | Mean ∆ (95% CI)^1^ |  | Estimate (95% CI)^2^ | Estimate (95% CI)^2^ | Estimate (95% CI)^2^ |
| Willingness-to-try-new-foods, n=786 | 0.07 (0.03, 0.11)** | 0.06 (0.01, 0.1)* | 0.02 (-0.03, 0.08) |  | 0.04 (-0.02, 0.11) | 0.03 (-0.04, 0.1) | -0.01 (-0.07, 0.05) |
| Number of fruits rated *yucky*, n=763 | 0.04 (-0.22, 0.31) | 0 (-0.33, 0.32) | -0.11 (-0.45, 0.24) |  | 0.15 (-0.28, 0.58) | 0.1 (-0.37, 0.58) | -0.05 (-0.46, 0.37) |
| Number of fruits rated *ok*, n =763 | -0.23 (-0.54, 0.07) | -0.05 (-0.42, 0.32) | 0.04 (-0.36, 0.44) |  | -0.27 (-0.77, 0.22) | -0.09 (-0.63, 0.45) | 0.18 (-0.3, 0.66) |
| Number of fruits rated *yummy*, n=763 | 0.4 (0.09, 0.72)* | 0.26 (-0.12, 0.65) | 0.18 (-0.23, 0.59) |  | 0.22 (-0.29, 0.74) | 0.09 (-0.48, 0.65) | -0.14 (-0.64, 0.36) |
| Number of vegetables rated *yucky*, n=763 | 0.25 (-0.11, 0.61) | 0.23 (-0.22, 0.67) | 0 (-0.48, 0.47) |  | 0.25 (-0.34, 0.85) | 0.23 (-0.42, 0.88) | -0.02 (-0.6, 0.55) |
| Number of vegetables rated *ok*, n=763 | -0.27 (-0.64, 0.09) | 0.01 (-0.44, 0.46) | 0.12 (-0.36, 0.59) |  | -0.39 (-0.99, 0.21) | -0.11 (-0.76, 0.55) | 0.28 (-0.29, 0.86) |
| Number of vegetables rated *yummy*, n=763 | 0.29 (-0.1, 0.67) | 0.03 (-0.45, 0.51) | 0.2 (-0.31, 0.71) |  | 0.09 (-0.55, 0.73) | -0.17 (-0.86, 0.53) | -0.26 (-0.87, 0.36) |
| Gross motor skill |  |  |  |  |  |  |  |
| Gross motor quotient^3^, n=254 | -2.6 (-6.42, 1.21) | -3.09 (-7.25, 1.06) | -7.91 (-12.01, -3.81)** |  | 5.31 (-0.29, 10.91) | 4.82 (-1.02, 10.65) | -0.49 (-6.13, 5.15) |
| Object control^3^, n=263 | -0.44 (-1.07, 0.18) | -0.26 (-0.96, 0.44) | -0.38 (-1.07, 0.31) |  | -0.06 (-0.99, 0.87) | 0.12 (-0.87, 1.1) | 0.18 (-0.76, 1.12) |
| Locomotor^3^, n=254 | -0.41 (-1.4, 0.57) | -0.71 (-1.78, 0.36) | -2.24 (-3.29, -1.18)** |  | 1.83 (0.39, 3.27)* | 1.53 (0.03, 3.04)* | -0.3 (-1.75, 1.16) |
| Physical activity |  |  |  |  |  |  |  |
| MVPA, n=713 | 16.84 (12.81, 20.87)** | 9.27 (4.26, 14.29)** | 11.47 (6.32, 16.62)** |  | 5.37 (-1.17, 11.91) | -2.2 (-9.39, 4.99) | -7.57 (-14, -1.13)* |
| LPA, n=713 | 1.14 (-5.59, 7.86) | 7.69 (-0.68, 16.06) | 7.08 (-1.5, 15.66) |  | -5.94 (-16.84, 4.96) | 0.61 (-11.38, 12.6) | 6.55 (-4.18, 17.28) |
| Sed/S, n=713 | -17.97 (-26.4, -9.54)** | -16.82 (-27.31, -6.32)** | -18.41 (-29.17, -7.65)** |  | 0.44 (-13.23, 14.11) | 1.59 (-13.44, 16.62) | 1.15 (-12.31, 14.61) |
| BMI-Z, n=851 | -0.04 (-0.08, 0.01) | -0.06 (-0.11, 0) | 0.04 (-0.02, 0.1) |  | -0.08 (-0.15, 0)* | -0.1 (-0.18, -0.02)* | -0.02 (-0.09, 0.05) |

Abbreviations: ∆, change; CI, confidence interval; MVPA, moderate to vigorous physical activity; LPA, light physical activity; Sed/S, sedentary time/sleep; BMIz, body mass index z-score.

^1^ Results from linear mixed models. Estimated values represent mean within group difference from baseline to endline. * p < 0.05; ** p < 0.01

^2^ Results from pairwise comparisons between study groups. Values represent mean between group differences in the change in score from baseline to endline. * p < 0.05; ** p < 0.01

^3^ 2017-2018 cohort only

Supplemental Table 5. Baseline to endline changes in objective child measures within and between study arms, selecting one child per family in the CHAMP cluster-randomized controlled trial

|  | **CHAMP** | **CHAMP+** | **Control** | **CHAMP vs Control** | **CHAMP+ vs Control** | **CHAMP+ vs CHAMP** |
| --- | --- | --- | --- | --- | --- | --- |
|  | Mean ∆ (95% CI)^1^ | Mean ∆ (95% CI)^1^ | Mean ∆ (95% CI)^1^ | Adjusted Estimate (95% CI)^2^ | Adjusted Estimate (95% CI)^2^ | Adjusted Estimate (95% CI)^2^ |
| Willingness-to-try-new-foods, n = 730 | 0.07 (0.03, 0.11)** | 0.06 (0.01, 0.11)* | 0.03 (-0.02, 0.09) | 0.04 (-0.03, 0.1) | 0.03 (-0.05, 0.1) | -0.01 (-0.07, 0.06) |
| Fruit and Vegetable Preference |  |  |  |  |  |  |
| Number of fruits rated *yucky*, n = 709 | 0.05 (-0.22, 0.32) | -0.06 (-0.39, 0.27) | -0.09 (-0.46, 0.27) | 0.14 (-0.31, 0.59) | 0.04 (-0.46, 0.53) | -0.11 (-0.54, 0.32) |
| Number of fruits rated *ok*, n = 709 | -0.26 (-0.57, 0.05) | 0.01 (-0.37, 0.39) | 0.02 (-0.39, 0.43) | -0.28 (-0.79, 0.23) | -0.01 (-0.57, 0.55) | 0.27 (-0.22, 0.76) |
| Number of fruits rated *yummy*, n = 709 | 0.41 (0.08, 0.73)* | 0.3 (-0.09, 0.7) | 0.13 (-0.3, 0.56) | 0.27 (-0.27, 0.81) | 0.17 (-0.42, 0.75) | -0.1 (-0.61, 0.41) |
| Number of vegetables rated *yucky*, n = 709 | 0.15 (-0.21, 0.52) | 0.15 (-0.3, 0.6) | -0.02 (-0.5, 0.47) | 0.17 (-0.44, 0.78) | 0.16 (-0.5, 0.83) | 0 (-0.58, 0.58) |
| Number of vegetables rated *ok*, n = 709 | -0.24 (-0.61, 0.12) | 0.09 (-0.36, 0.54) | 0.1 (-0.38, 0.59) | -0.34 (-0.95, 0.26) | -0.01 (-0.67, 0.65) | 0.33 (-0.25, 0.91) |
| Number of vegetables rated *yummy*, n = 709 | 0.32 (-0.08, 0.71) | 0.09 (-0.4, 0.58) | 0.15 (-0.37, 0.68) | 0.16 (-0.5, 0.82) | -0.06 (-0.78, 0.66) | -0.22 (-0.85, 0.41) |
| Gross Motor Skill |  |  |  |  |  |  |
| Gross motor quotient^3^, n = 229 | -1.76 (-5.8, 2.29) | -2.41 (-6.78, 1.96) | -7.09 (-11.29, -2.89)** | 5.33 (-0.5, 11.17) | 4.68 (-1.37, 10.74) | -0.65 (-6.6, 5.31) |
| Object control^3^, n = 237 | -0.45 (-1.1, 0.21) | -0.12 (-0.86, 0.62) | -0.38 (-1.09, 0.33) | -0.07 (-1.04, 0.9) | 0.26 (-0.76, 1.29) | 0.33 (-0.66, 1.32) |
| Locomotor^3^, n = 229 | -0.11 (-1.15, 0.94) | -0.67 (-1.8, 0.46) | -1.98 (-3.06, -0.9)** | 1.87 (0.37, 3.37)* | 1.31 (-0.25, 2.87) | -0.57 (-2.1, 0.97) |
| Physical Activity |  |  |  |  |  |  |
| MVPA, n = 658 | 16.85 (12.6, 21.09)** | 8.86 (3.57, 14.15)** | 11.17 (5.65, 16.69)** | 5.67 (-1.29, 12.63) | -2.31 (-9.96, 5.33) | -7.99 (-14.76, -1.21)* |
| LPA, n = 658 | 1.13 (-5.77, 8.02) | 6.98 (-1.63, 15.6) | 5.62 (-3.35, 14.59) | -4.49 (-15.8, 6.82) | 1.37 (-11.07, 13.8) | 5.86 (-5.18, 16.89) |
| Sed/S, n = 658 | -17.91 (-26.59, -9.23)** | -15.74 (-26.58, -4.9)** | -16.74 (-28.02, -5.45)** | -1.17 (-15.41, 13.06) | 1 (-14.65, 16.65) | 2.17 (-11.71, 16.06) |
| BMIz, n = 782 | -0.03 (-0.08, 0.01) | -0.06 (-0.12, 0)* | 0.03 (-0.04, 0.09) | -0.06 (-0.14, 0.02) | -0.09 (-0.17, 0) | -0.03 (-0.1, 0.05) |

Abbreviations: ∆, change; CI, confidence interval; MVPA, moderate to vigorous physical activity; LPA, light physical activity; Sed/S, sedentary time/sleep; BMIz, body mass index z-score.

^1^ Results from linear mixed models adjusted for child age and race and ethnicity. Estimated values represent adjusted mean within group difference from baseline to endline. * p < 0.05; ** p < 0.01

^2^ Results from pairwise comparisons between study groups. Values represent mean between group differences in the change in score from baseline to endline. * p < 0.05; ** p < 0.01

^3^ 2017-2018 cohort only

Supplemental Table 6. Center and subject intraclass correlations for objective child measures in the CHAMP cluster-randomized controlled trial^1^

|  | **ICC for center** | **ICC for subjects** |
| --- | --- | --- |
| Willingness-to-try-new-foods, n = 757 | 0.007 | 0.619 |
| Fruit and Vegetable Preference |  |  |
| Number of fruits rated *yucky*, n = 735 | 0.018 | 0.257 |
| Number of fruits rated *ok*, n = 735 | 0.024 | 0.173 |
| Number of fruits rated *yummy*, n = 735 | 0.030 | 0.368 |
| Number of vegetables rated *yucky*, n = 735 | 0.030 | 0.446 |
| Number of vegetables rated *ok*, n = 735 | 0 | 0.155 |
| Number of vegetables rated *yummy*, n = 735 | 0.016 | 0.448 |
| Gross Motor Skill |  |  |
| Gross motor quotient^3^, n = 237 | 0.012 | 0.308 |
| Object control^3^, n = 245 | 0.049 | 0.292 |
| Locomotor^3^, n = 237 | 0.044 | 0.105 |
| Physical Activity |  |  |
| MVPA, n = 685 | 0.064 | 0.455 |
| LPA, n = 685 | 0.061 | 0.519 |
| Sed/S, n = 685 | 0.042 | 0.544 |
| BMIz, n = 812 | 0.009 | 0.927 |

Abbreviations: ICC, intraclass correlation; MVPA, moderate to vigorous physical activity; LPA, light physical activity; Sed/S, sedentary time/sleep; BMIz, body mass index z-score.

^1^ Results from linear mixed models adjusted for child age and race and ethnicity.

Supplemental Table 7. Independent associations of data collector with locomotor and object control scores by cohort in the CHAMP cluster-randomized controlled trial

|  | 2017-2018 Cohort |  | 2018-2019 Cohort |
| --- | --- | --- | --- |
|  | Type III ANOVA p^1^ |  | Type III ANOVA p^1^ |
| Locomotor | 0.18 |  | <0.01** |
| Object control | 0.61 |  | <0.01** |

Abbreviations: ANOVA, analysis of variance

^1^ Results from linear mixed models adjusted for child age and race and ethnicity. P values represent type III analysis of variance for data collector x time interaction. * p < 0.05; ** p < 0.01
